# A meta-analysis of genetic and phenotypic diversity of European local pig breeds reveals genomic regions associated with breed differentiation for production traits

**DOI:** 10.1186/s12711-023-00858-3

**Published:** 2023-12-07

**Authors:** Klavdija Poklukar, Camille Mestre, Martin Škrlep, Marjeta Čandek-Potokar, Cristina Ovilo, Luca Fontanesi, Juliette Riquet, Samuele Bovo, Giuseppina Schiavo, Anisa Ribani, Maria Muñoz, Maurizio Gallo, Ricardo Bozzi, Rui Charneca, Raquel Quintanilla, Goran Kušec, Marie-José Mercat, Christoph Zimmer, Violeta Razmaite, Jose P. Araujo, Čedomir Radović, Radomir Savić, Danijel Karolyi, Bertrand Servin

**Affiliations:** 1 Agricultural Institute of Slovenia, Hacquetova Ulica 17, 1000 Ljubljana, Slovenia; 2 GenPhySE, Université de Toulouse, INRAE, INP, ENVT, 31320 Castanet-Tolosan, France; 3 Departamento Mejora Genética Animal, INIA-CSIC, Crta. de la Coruña Km. 7,5, 28040 Madrid, Spain; 4 Department of Agricultural and Food Sciences, Division of Animal Sciences, University of Bologna, Viale Fanin 46, 40127 Bologna, Italy; 5 Associazione Nazionale Allevatori Suini (ANAS), Via Nizza 53, 00198 Rome, Italy; 6 DAGRI-Animal Science Section, Università Di Firenze, Via Delle Cascine 5, 50144 Florence, Italy; 7 MED- Mediterranean Institute for Agriculture, Environment and Development, Universidade de Évora, Pólo da Mitra, Apartado 94, 7006-554 Évora, Portugal; 8 Programa de Genética y Mejora Animal, IRTA, Torre Marimon, Caldes de Montbui, 08140 Barcelona, Spain; 9 Faculty of Agrobiotechnical Sciences, University of Osijek, Vladimira Preloga 1, 31000 Osijek, Croatia; 10 IFIP Institut du Porc, La Motte au Vicomte, BP 35104, 35651 Le Rheu Cedex, France; 11 Bauerliche Erzeugergemeinschaft Schwäbisch Hall, Haller Str. 20, 74549 Wolpertshausen, Germany; 12 Animal Science Institute, Lithuanian University of Health Sciences, 82317 Baisogala, Lithuania; 13 Centro de Investigação de Montanha (CIMO), Instituto Politécnico de Viana do Castelo, Escola Superior Agrária, Refóios do Lima, 4990-706 Ponte de Lima, Portugal; 14 Department of Pig Breeding and Genetics, Institute for Animal Husbandry, 11080 Belgrade-Zemun, Serbia; 15 Faculty of Agriculture, University of Belgrade, Nemanjina 6, 11080 Belgrade-Zemun, Serbia; 16 Department of Animal Science, Faculty of Agriculture, University of Zagreb, Svetošimunska c. 25, 10000 Zagreb, Croatia

## Abstract

**Background:**

Intense selection of modern pig breeds has resulted in genetic improvement of production traits while the performance of local pig breeds has remained lower. As local pig breeds have been bred in extensive systems, they have adapted to specific environmental conditions, resulting in a rich genotypic and phenotypic diversity. This study is based on European local pig breeds that have been genetically characterized using DNA-pool sequencing data and phenotypically characterized using breed level phenotypes related to stature, fatness, growth, and reproductive performance traits. These data were analyzed using a dedicated approach to detect signatures of selection linked to phenotypic traits in order to uncover potential candidate genes that may underlie adaptation to specific environments.

**Results:**

Analysis of the genetic data of European pig breeds revealed four main axes of genetic variation represented by the Iberian and three modern breeds (i.e. Large White, Landrace, and Duroc). In addition, breeds clustered according to their geographical origin, for example French Gascon and Basque breeds, Italian Apulo Calabrese and Casertana breeds, Spanish Iberian, and Portuguese Alentejano breeds. Principal component analysis of the phenotypic data distinguished the larger and leaner breeds with better growth potential and reproductive performance from the smaller and fatter breeds with low growth and reproductive efficiency. Linking the signatures of selection with phenotype identified 16 significant genomic regions associated with stature, 24 with fatness, 2 with growth, and 192 with reproduction. Among them, several regions contained candidate genes with possible biological effects on stature, fatness, growth, and reproductive performance traits. For example, strong associations were found for stature in two regions containing, respectively, the *ANXA4* and *ANTXR1* genes, for fatness in a region containing the *DNMT3A* and *POMC* genes and for reproductive performance in a region containing the *HSD17B7* gene.

**Conclusions:**

In this study on European local pig breeds, we used a dedicated approach for detecting signatures of selection that were supported by phenotypic data at the breed level to identify potential candidate genes that may have adapted to different living environments and production systems.

**Supplementary Information:**

The online version contains supplementary material available at 10.1186/s12711-023-00858-3.

## Background

In the last decades, pig breeding has focused mainly on improving growth rate, carcass leanness, and reproductive performance [1] of a limited number of breeds [2]. In parallel, most local breeds have not been subjected to such intensive management or genetic improvement and their use has declined. Local breeds are often raised in extensive farming systems, resulting in adaptation to specific environmental conditions and (often) poor feeding resources [3]. However, this adaptation to seasonal fluctuations in feed availability may have resulted in low productivity [4]. As a result, many local pig breeds have been abandoned or have even become extinct, while most of them have faced population bottlenecks and genetic drift or introgression from other pig populations [5, 6].

Today, many local breeds are used on a relatively limited scale and the information on their phenotypic traits and genetic diversity is available only for a few of them, such as the Iberian and the Meishan breeds [7]. Nonetheless, interest in local pig breeds has recently increased for several reasons, including their meat quality (allowing the production of high-quality meat products), their adaptation to local feeding resources, and society's awareness of the need to conserve phenotypic and genetic biodiversity [3]. Since local pig breeds are exposed to specific selection pressures in different local environments, they also represent interesting genetic resources [8] that could become more important in the future as a reservoir of genetic diversity to adapt to global change.

Recent genetic characterization of 20 European local pig breeds showed that some local breeds are clustered according to their geographical distribution (e.g. French Gascon and Basque breeds, Italian Apulo Calabrese and Casertana breeds, Spanish Iberian and Portuguese Alentejano breed), while others suffer from introgression or admixture with modern pig breeds (e.g. Lietuvos Baltosios Senojo Tipo and Lietuvos Vietiné with Large White and Landrace pigs; and Mora Romagnola with Duroc pig) [8–10]. Consequently, these breeds have developed particular phenotypic traits that could reflect specific genetic potential and adaptation to different production systems. As the measurement of phenotypic traits in pigs is to some extent standardized, it is possible to compare local breeds. As shown in the study of Čandek-Potokar and Nieto [3] who reviewed production traits in various breeds, not only do European local pig breeds differ from modern breeds, but they also exhibit extensive variation between them, and their phenotypes reflect the heterogeneity of production systems and management of local breeds.

To better understand the genetic basis that underlies variation in phenotypic traits of local pig breeds, several genome-wide association studies have focused on detecting associations of loci with different phenotypic attributes, such as morphological, production, or meat quality traits [11–14]. However, in these studies the sample sizes were small, which increases the risk to obtain false negative results due to low statistical power [15]. Another approach to search for associations between genetic polymorphisms and phenotypes is to look for genomic regions that have responded to selection (i.e., signatures of selection). Several studies in local breeds have shown that the signals detected contain gene variants/genes that may be associated with variation in phenotype, such as coat color, growth, reproduction, or fatness [8, 9, 16–19]. The study by Muñoz et al. [9] identified putative signatures of selection using single nucleotide polymorphism (SNP)-array data from 20 European local breeds for regions containing genes involved in fatness, growth, reproduction, development, behavior and sensory perception. To increase the precision of the localization of selection signals, whole-genome sequencing was performed on pooled DNA samples from the same breeds/animals, and new analyses detected several regions that were associated with coat color, body size, growth, reproduction, and fat deposition [8]. These two studies exploited methods that are based on the differences in allele frequencies between breeds to detect selection signals. Other approaches have been proposed that jointly exploit information on allele frequencies and population-level phenotypes [20, 21], which can increase the power to detect adaptation specific to some traits or environmental factors. In the current study, we adapted such an approach to combine the DNA-pool sequencing data from 19 European local [8] and seven populations of modern breeds and the database of many phenotypic traits in 20 local pig breeds associated with stature, fatness, growth, and reproductive performance from [3], in order to identify additional signatures of selection that are associated with specific breed level phenotypes and to provide hypotheses on the physiological processes involved in genetic divergence between local breeds.

## Methods

The aim of this study was to localize genomic regions associated with signatures of selection of local pig breeds for production traits by combining genetic and phenotypic data at the breed (population) level. Most of the data used for this study have been previously published [3, 8, 9]. In this section, the genetic and phenotypic datasets are presented, followed by a description of the methodological approach that was used to detect signatures of selection on phenotypic traits.

### Genetic datasets

The current study was based on genetic data collected from 19 populations of European local pig breeds and seven populations of modern breeds. The dataset included SNP genotyping data obtained with a medium-density array [9] for 20 local breeds and whole-genome sequencing data of the pooled samples [8] for all these breeds, except the Iberian breed. To better describe the genetic structure of the local pig breeds, samples from seven additional populations of four modern pig breeds were added. The final collection of breeds used for this study is shown in Table 1. A summary of the whole-genome sequencing statistics was previously described in [8] and is presented also in Additional file 1: Table S1.

**Table 1:** Name and country of origin of the pig breeds included in this study

Pig breed name	Country	Number of individuals genotyped on the SNP array	Sample size for the DNA-pool sequencing
Local breeds			
Alentejano	Portugal	48	35
Apulo Calabrese	Italy	53	35
Basque	France	39	30
Bísaro	Portugal	48	35
Black Slavonian	Croatia	52	35
Cinta Senese	Italy	51	35
Gascon	France	48	30
Iberian	Spain	48	–
Krškopolje	Slovenia	52	35
Lietuvos Baltosios Senojo Tipo	Lithuania	48	35
Lietuvos Vietiné	Lithuania	48	35
Mangalitsa	Serbia	50	35
Mora Romagnola	Italy	48	35
Moravka	Serbia	49	35
Negre Mallorquí	Spain	48	35
Nero Casertano	Italy	53	35
Nero Siciliano	Italy	48	35
Sarda	Italy	48	35
Schwäbisch-Hällisches	Germany	49	35
Turopolje	Croatia	49	35
Modern breeds			
Italian Large White	Italy	4	35
Large White	France	97	–
Italian Duroc	Italy	5	35
Duroc	France	33	–
Italian Landrace	Italy	4	35
Landrace	France	53	–
Pietrain	France	61	–

### Quality control of the genetic datasets

Quality control of the SNP genotypes from the array was performed for the entire dataset using standard filters: only autosomal SNPs with less than 10% missing data were retained. Following this step, 10 individuals with more than 3% missing genotypes were clear outliers and were thus discarded.

Discovery of de novo SNPs was carried out on DNA-pool sequencing data using the CRISP software [22] with default parameters, which yielded 34,751,691 variants. From these, we filtered out variants with more than two alleles, those that most likely resulted from sequencing errors based on a very low minor allele frequency (field VP = 0 and AF = 1), and those with a low mapping quality (QUAL < 1000, MQ < 20). After filtering, 16,403,270 SNPs remained and the allele frequencies were estimated for all them.

### Genetic structure of the pig populations

The genetic structure of the pig populations and the covariance between the populations were assessed from the individual SNP genotyping data using principal component analysis (PCA), admixture analysis [23], and population tree reconstruction using the hapFLK software [24]. Admixture analysis was performed for the number of clusters (K) between 2 to 40 to determine the value of K that best explained the data. The decrease in cross-validation error was monotonous from 2 to 40 (Additional file 2: Fig. S1), but showed a diminishing decrease after K = 24 and, therefore, this value was used as a reference to describe the genetic structure of the breeds. Based on the admixture results, individuals that had more than 80% of their genome assigned to the main cluster of their assigned breed were selected for inclusion in the population tree analysis. The Nero Siciliano breed did not have any individual that met this criterion and was, therefore, not included in the population tree reconstruction.

To verify the quality of the DNA-pool sequence data, we compared these data with the SNP array genotyping results. Allele frequencies of the array SNPs in the DNA-pool sequence data were estimated using allele counts extracted with the Samtools mpileup [25] and PoPoolation2 [26] software. Samtools was run with options -C 50 -q 20 (variants with a mapping quality less than 20 were discarded, as recommended by the software documentation). PoPoolation2 was run with default parameters on the resulting mpileup file. From the resulting pool of allele counts, allele frequencies and Fst for each pair of populations were calculated using the approach of [27] as implemented in the R package poolfstat. The population tree was constructed by applying the neighbour-joining algorithm on the Fst matrix, using the same procedure as used for allele frequencies derived from the SNP array genotypes.

### Phenotypic characterization of local pig populations

A database of phenotypic traits of European local pig populations [3] was used to determine global differences in phenotype between the breeds. It contains the results of different studies in which the main experimental unit for the phenotypic trait was a trial, an experiment, or part of an experiment in which rearing conditions were sometimes very different from usual production conditions. The availability and quality of data were breed-dependent; for certain breeds (e.g. Iberian and Alentejano), the data were more exhaustive than for some less studied breeds. The collection of data on the traits/variables considered here was standardized, as described in Additional file 1: Table S2. In many cases, age was not available and, therefore, live weight was considered (e.g. growth performance standardization and backfat thickness adjustment). Phenotypic variables were combined into four distinct groups that summarized stature, fatness, growth, and reproductive performance. The growth performance group included average daily gain records for three growth periods, during which the animals were fed ad libitum (i.e., from lactation to the early fattening phase; up to approximately 60 kg). The stature group included body weight and height records on adult male and female animals. The fatness group included backfat thickness records at different anatomical locations, fatty acid composition, carcass lean meat content, and intramuscular fat content. The reproductive performance group included number of piglets per litter, number of litters per year, piglets/litter weights, duration of lactation, and farrowing interval. A more detailed description of the traits included in the phenotypic characterization is in Additional file 1: Table S2.

The means for each variable and each breed were calculated and scaled. Since the phenotypic database was composed of results from different studies, some variables were missing for some breeds (Additional file 2: Fig. S2). Missing data were imputed with a regularized iterative PCA method using the R package missMDA [28]. The imputed mean values for each trait, together with information on the number of experimental units considered, are in Additional file 3: Tables S3 and 4. The experimental units that were extracted from all studies were given equal weight regardless of the number of pigs involved. Principal component analyses were performed using the R package FactoMiner [29] for growth performance, reproductive performance, stature, and fatness traits. Uncertainty in the predictions of missing data was assessed by multiple imputation (MIPCA function [30], Additional file 2: Fig. S3). Breed loadings on the first PC for each PCA were used as “breed scores” for the analysis of signatures of selection. All statistical analyses were performed using the R statistical software.

### Genome scan for selection on phenotypic breed scores

To identify genomic regions that are associated with selection on breed level phenotypes, we built upon the approach of [20], which we briefly describe here. Assuming data on allele frequencies measured in $\mathrm{r}$ populations at $\mathrm{L}$ loci, the association model at a locus $\mathrm{l}$ is:

1 $${\bf{p}}^{1} \; \sim \;{\text{N}}\left( {{\bf{1}}{\text{p}}_{0}^{1} \;{ + }\;{\bf{x}}{\upbeta }^{1} {,}{\bf{V}}{\text{p}}_{0}^{1} \left( {{1}\; - \;{\text{p}}_{0}^{1} } \right)} \right)$$

where ${\mathbf{p}}^{\mathrm{l}}$ is the vector of allele frequencies in the $\mathrm{r}$ populations, ${\mathrm{p}}_{0}^{\mathrm{l}}$ is the (unknown) ancestral allele frequency of the locus, $\mathbf{x}$ is the vector of breed level phenotypes, ${\upbeta }^{\mathrm{l}}$ is the effect (regression coefficient) of phenotype on differences in allele frequencies, and $\mathbf{V}$ is the genome-wide variance–covariance matrix of allele frequencies between populations. The idea behind this model is that adaptation of populations to covariate $\mathbf{x}$ (here the breed level phenotypes) drives differences in allele frequency between populations away from their expectation under genetic drift. In Model (1), this expectation is modelled with the genome-wide covariance matrix $\mathbf{V}$ (see [20] for a detailed description). To perform statistical inference under this model, the parameters $\mathbf{V}$, ${\upbeta }^{\mathrm{l}}$, and ${\mathrm{p}}_{0}^{\mathrm{l}}$ need to be estimated. Under the null hypothesis (i.e. selection associated with phenotype $\mathrm{x}$ has not affected the allele frequencies), parameter ${\upbeta }^{\mathrm{l}}$ is set to 0. To test for association with the covariate $\mathbf{x}$, Coop et al. [20] used a Monte Carlo Markov chain (MCMC) algorithm, which allows derivation of a statistic for association (a Bayes factor). This was later extended by [21] to account for uncertainty in allele frequency estimation in DNA-pool sequencing experiments. This approach was tested on our dataset, but was found computationally inefficient due to the very large number of SNPs considered. Thus, we used a frequentist treatment of the model that consists of maximizing the likelihood of Model (1) under the null and the alternative hypotheses and performing a likelihood ratio test. One deviation from this approach is that variation in sequencing depth between DNA-pools was accounted for by using regularized allele frequencies ${\widetilde{\mathrm{p}}}_{\mathrm{r}}^{\mathrm{l}}$ (see below) rather than fitting the model on the usual allele frequency estimates ${\widehat{\mathrm{p}}}_{\mathrm{r}}^{\mathrm{l}}$. The maximum likelihood estimator of allele frequencies is ${\widehat{\mathrm{p}}}_{\mathrm{r}}^{\mathrm{l}}=\frac{{\mathrm{c}}_{\mathrm{r}}^{\mathrm{l}}}{{\mathrm{n}}_{\mathrm{r}}^{\mathrm{l}}}$, where ${\mathrm{c}}_{\mathrm{r}}^{\mathrm{l}}$ is the count of alternative alleles and ${\mathrm{n}}_{\mathrm{r}}^{\mathrm{l}}$ the sequencing depth for population $\mathrm{r}$ at locus $\mathrm{l}$. This estimator has good properties provided the sequencing depth ${\mathrm{n}}_{\mathrm{r}}^{\mathrm{l}}$ at the locus is high. However, sequencing depth was highly variable along the genome and was quite low (even 0) in some genomic regions. Moreover, it varies between populations at a given locus. Thus, to regularize allele frequencies estimates, we used shrunk allele frequencies estimates as follows:

$${\widetilde{\mathrm{p}}}_{\mathrm{r}}^{\mathrm{l}}=\frac{{\mathrm{a}}^{\mathrm{l}}+{\mathrm{c}}_{\mathrm{r}}^{\mathrm{l}}}{{\mathrm{b}}^{\mathrm{l}}+{\mathrm{n}}_{\mathrm{r}}^{\mathrm{l}}},$$

where ${\mathrm{a}}^{\mathrm{l}}$ and ${\mathrm{b}}^{\mathrm{l}}$ are regularizing (prior) parameters set such that ${\mathrm{a}}^{\mathrm{l}}/{\mathrm{b}}^{\mathrm{l}}$ equals the frequency of the alternative allele at locus $\mathrm{l}$ among all populations. Thus, if a population has no observed data at a SNP, its allele frequency will be similar to that in other populations, which reduces the risk of false positives due to uneven sequencing coverage.

The association of breed scores to allele frequencies was tested for all SNPs for all breed scores (stature, growth performance, fatness traits, and reproductive performance). The result of the test was an asymptotic p-value for each SNP for each phenotypic trait. Since Model (1) is only valid for intermediate ancestral allele frequencies, only SNPs for which the ancestral allele frequency estimate, ${\mathrm{p}}_{0}^{\mathrm{l}},$ was between 0.05 and 0.95 were considered in the following. To reduce the possibility of false positives, a permutation procedure was conducted to correct the asymptotic p-values. For each phenotype tested, a new analysis was performed on a permuted phenotype data set, *i.e.* under the null hypothesis of no association between the phenotype and the allele frequencies. This produced an empirical distribution of the asymptotic p-values under the null hypothesis that was used to obtain corrected p-values at each SNP. Statistical significance was then established on the corrected p-values by estimating the false discovery rates (FDR) with the approach of [31], using the qvalue R package. The FDR threshold for detecting significant associations was set at 1%.

Based on association statistics for individual SNPs, we identified regions of association and extracted the candidate genes from the annotation of the reference genome (*Sscrofa11.1*). Regions that contained at least four significant SNPs less than 200 kb apart were further annotated. The detected genes within these regions were reviewed in the literature for potential biological effects on phenotypic differentiation.

## Results

### Genetic structure of European pig breeds

**Fig. 1:**
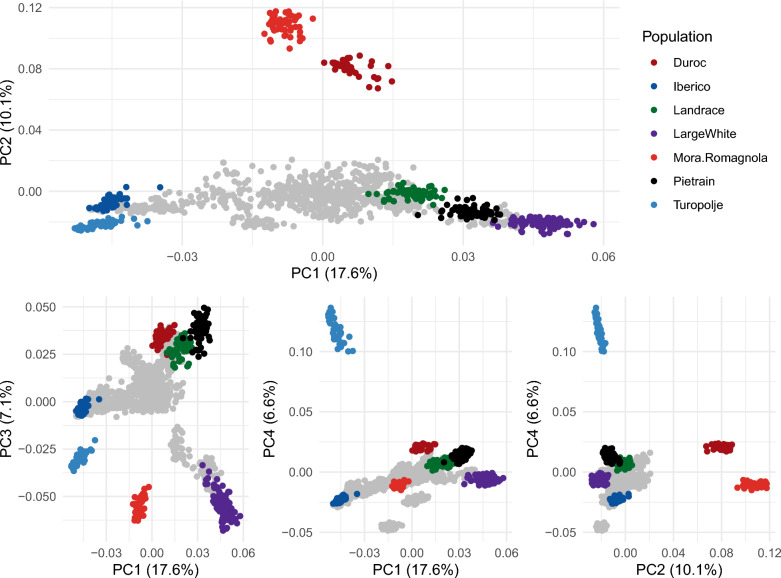
Principal component analysis of 24 pig breeds genotyped using a medium-density SNP array

To characterize the genetic structure of populations, the individual genotypes on the SNP array were used to perform a standard genetic structure analysis. PCA analyses of 24 breeds revealed the main genetic backgrounds present in the dataset (i.e. Iberian, Duroc/Mora Romagnola, Large White, and Landrace/Pietrain), which were visible in the first three principal components (Fig. 1). PC1 and PC2 clearly distinguished between the Iberian pig, White pigs (Landrace, Large White, and Pietrain) and Duroc/Mora Romagnola backgrounds. PC3 separated the White pigs’ background into a Large White background and a Landrace/Pietrain background. PC4 further separated the Turopolje breed from the Iberian group. The global pattern of differentiation between the breeds in our dataset is thus strongly influenced by modern pig breeds. Local pig breeds are usually at intermediate positions, mostly within a triangle with summits corresponding to the Iberian breed, the Landrace/Pietrain, and the Large White breeds (see Additional file 4: Fig. S12). The two exceptions are the Mora Romagnola and Turopolje breeds, which appear much more differentiated. In order to interpret these patterns, further analyses using admixture clustering and population tree reconstruction were performed.

**Fig. 2:**
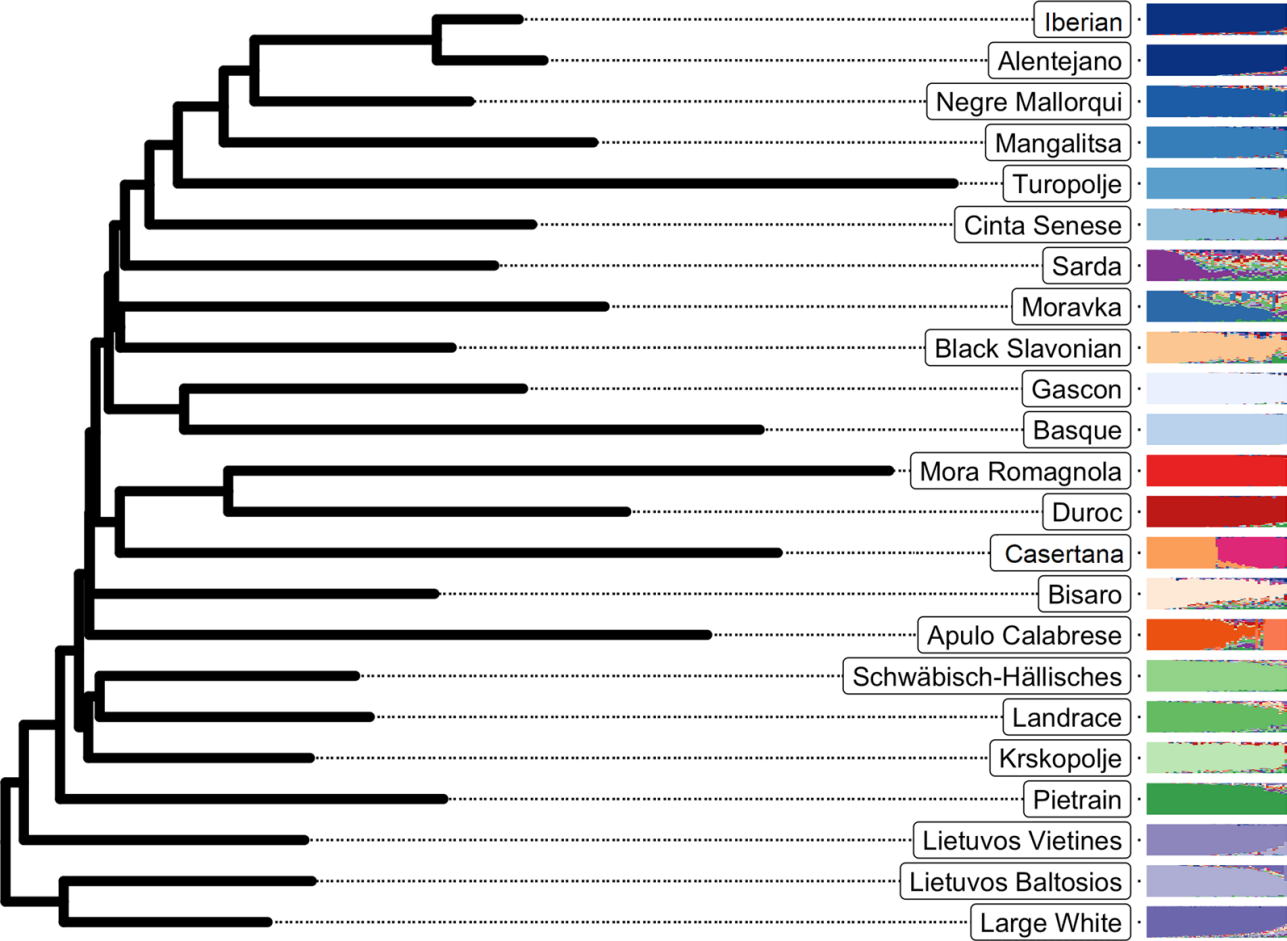
Population tree and admixture analysis of 23 pig breeds genotyped using a medium-density SNP array. The population tree constructed from pairwise genetic distances (Fst) is shown on the left, and the admixture component for all individuals belonging to the breed is shown on the right. The color levels follow the global axes of genetic variation, with populations most closely related to the Iberian type shown in shades of blue, to the Duroc in shades of red, to the Large White in shades of purple, and to the Landrace in shades of green. Heterogeneous populations, or those equidistant from these four clusters, are shown in orange. The Nero Siciliano breed exhibited extremely high heterogeneity and was, therefore, not included in the reconstruction of the population tree and is not shown

The results of the admixture analysis show that the number of homogeneous clusters in the dataset is difficult to determine. The cross-validation procedure was conducted for up to 40 clusters and resulted in a general decrease in cross-validation error with an increasing number of clusters (see Additional file 2: Fig. S1). However, the decrease slowed down after K = 24 clusters, a number that corresponds to the number of named breeds (Table 1). The further decrease beyond K = 24 was due to the fact that some breeds are sub-structured and required additional clusters to be well fitted. This is typical of local breeds (e.g. [32]). In the following, we present the results obtained with K = 24, which corresponds to the inflexion point in the cross-validation curve. Figure 2 shows the result of the population tree reconstruction and the admixture analysis side-by-side. At K = 24, most breeds exhibited a homogeneous pattern of admixture and belonged to a specific cluster, with the exceptions of the Alentejano and Iberian breeds (which belonged to the same cluster, in agreement with their common origin), the Casertana and Apulo Calabrese breeds (each of them further split into two groups), and the Sarda, Moravka, Bisaro, and Nero Siciliano breeds, which showed high heterogeneity. In the latter breeds, admixed individuals did not appear to be recent hybrids with other breeds. Admixture plots with K = 20, K = 15, K = 10, and K = 6 are shown using a circular plot in Additional file 2: Fig. S4.

The population tree was structured consistently with the main axes of variation identified in the PCA analysis for the main genetic backgrounds (Fig. 2). The Turopolje and Mora Romagnola breeds differentiated early in the PCA analysis, which could be explained based on results from the population analysis and the fact that they exhibited long branches corresponding to low heterozygosity. This analysis also revealed a general clustering of local breeds according to their geographical origin. Breeds closer to the Iberian group mostly originate from the Iberian peninsula or close geographical areas (South West of France for the Basque and Gascon breeds and Balearic Islands for the Negre Mallorqui breed). Interestingly, some other breeds from different geographical areas appeared to be related to this background, such as the Mangalitsa and Moravka from Serbia, the Black Slavonian from Croatia, and the Cinta Senese from Italy. Breeds from Central Europe, such as the Schwabisch-Hällisches and Krškopolje breeds, showed genetic proximity to the Landrace/Pietrain background, while breeds from Lithuania in Northern Europe showed genetic proximity with the Large White component. Finally, some breeds could not be considered to be related to any other breed in the dataset, such as the Bísaro from Portugal and the Apulo Calabrese from Italy.

Sequences from the investigated breeds were aligned to the Sscrofa11.1 reference genome and SNPs were discovered with the pool-seq variant caller CRISP. For SNPs on the genotyping array, allele frequencies were consistent with those derived from individual genotyping [9] (Additional file 2: Fig. S5). This confirmed the quality of the SNPs obtained by DNA-pool sequencing.

### Phenotypic differentiation of European local pig breeds

**Fig. 3:**
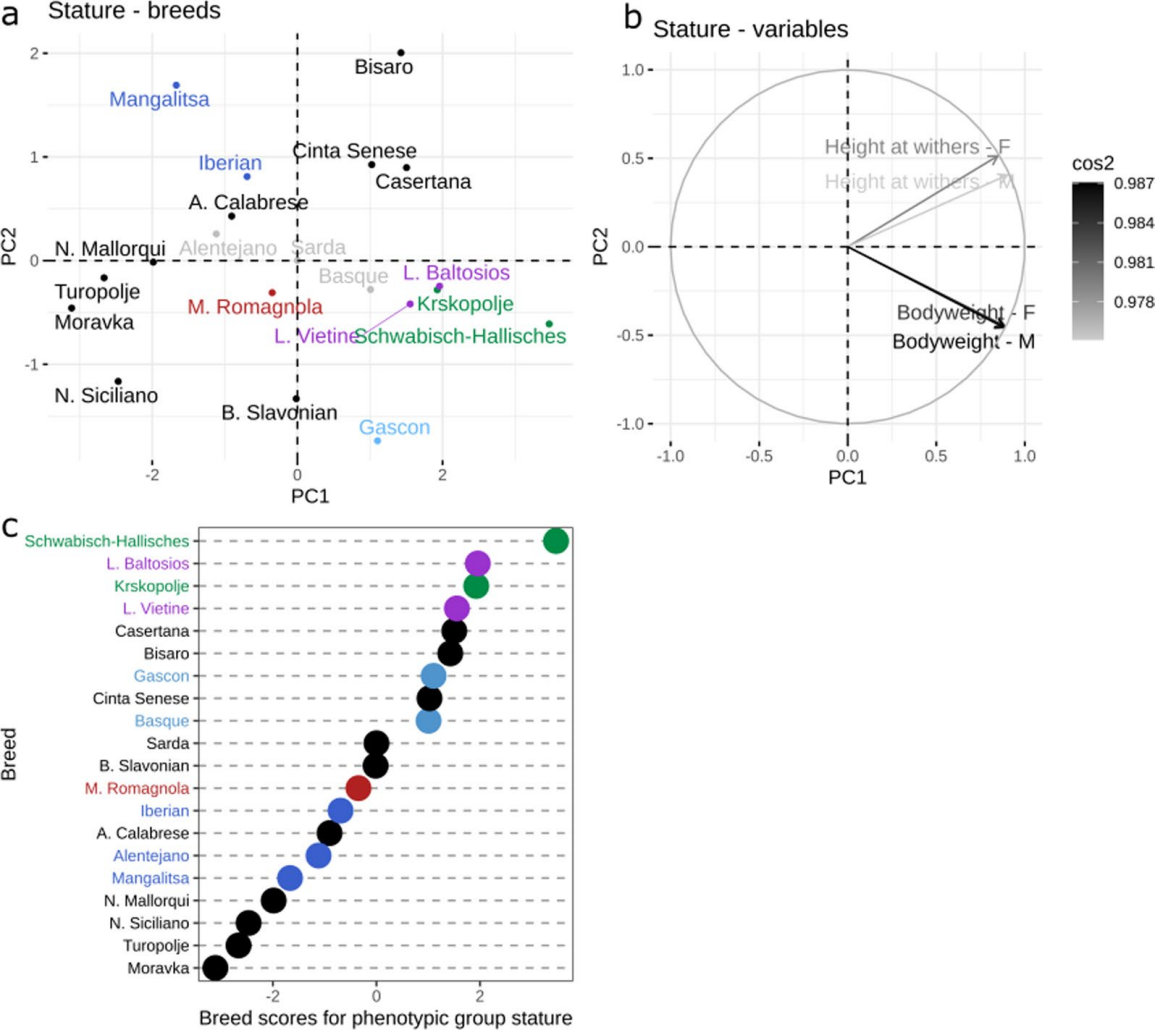
Principal component analysis showing the relationship of breeds (**a**) with traits associated with stature (**b**) and the corresponding phenotypic breed scores (**c**). Breeds (**a**) colored in grey are the breeds with more than 50% of missing variables and, thus, their position on the PCA must be interpreted carefully. The variables (**b**) are colored according to quality of the representation, which is measured by squared cosine between the vector originating from the element and its projection on the axis. The variables that contribute most to the separation of the trait into PC1 and PC2 are colored black. Breeds (**a** and **c**) are colored according to their genetic similarity. Breeds (**a** and **c**) in green are genetically Landrace-like breeds, in purple Large White-like breeds, in blue Iberian-like breeds, in red Duroc-like breeds, and in light blue the Gascon and Basque breeds

A database of published results on phenotypic traits of 20 local pig breeds [3] was used to distribute local pig populations according to phenotype, including stature, growth, fatness traits, and reproductive performance traits. The resulting relationships between breeds and variables was evaluated using PCA analyses. The first two principal components of the PCA for stature, growth, fatness, and reproduction group accounted for 98.2, 83.2, 84.3 and 70.7% of the total variance, respectively (Fig. 3) and (see Additional file 2 Figures S6–8). Scores for each breed from the PCA of each phenotypic group were extracted.

The first principal component for the stature group (Fig. 3) represented most of the total variability (77.3%) and clearly distributed the breeds according to average height and weight. Thus, breeds with lower PCA scores for stature (e.g. Moravka, Turopolje, Nero Siciliano, Negre Mallorqui) were smaller and lighter, while breeds with higher scores were taller and heavier breeds (e.g. Schwäbisch-Hällisches, Lietuvos Baltosios Senojo Tipo, Krškopolje pig, Lietuvos Vietiné).

The growth group distributed local breeds according to their growth capacity, including on average daily gain from weaning to the early fattening phase (i.e. up to 60 kg body weight) (Additional file 2: Fig. S6). The first PC explained 56.1% of the total variability and distributed the breeds with the highest (i.e. Schwäbisch-Hällisches, Lietuvos Baltosios Senojo Tipo, Lietuvos Vietiné, Bisaro, Krškopolje pig) and the lowest (i.e. Alentejano, Moravka, Black Slavonian, Mangalitsa and Turopolje) growth potential.

Fatness traits comprised traits associated with the fatty phenotype (Additional file 2: Fig. S7). Principal component 1 (59.3% of total variability) was positively correlated with backfat thickness at different anatomical locations and with intramuscular fat content. Conversely, lean meat and polyunsaturated fatty acid (PUFA) content (i.e., lean phenotype) were negatively correlated with PC1. Because intramuscular fat content is a trait of particular interest, the distribution of breeds by intramuscular fat content is shown in Additional file 2: Fig. S9. Local breeds were divided into fatter (e.g. Moravka, Iberian, Mangalitsa, Negre Mallorqui) and leaner phenotypes (e.g. Schwäbisch-Hällisches, Lietuvos Baltosios Senojo Tipo, Bisaro).

The final characterization of the local pig breeds was based on reproductive performance (Additional file 2: Fig. S8). Here, PC1 clearly distinguished breeds with larger litter sizes and greater birth weights (i.e. Schwäbisch-Hällisches, Lietuvos Baltosios Senojo Tipo, Lietuvos Vietiné, Bisaro, Krškopolje pig) from breeds that had lower reproductive performance, with smaller litters and lighter birth weights (i.e. Nero Siciliano, Turopolje, Casertana, Mora Romagnola).

**Fig. 4:**
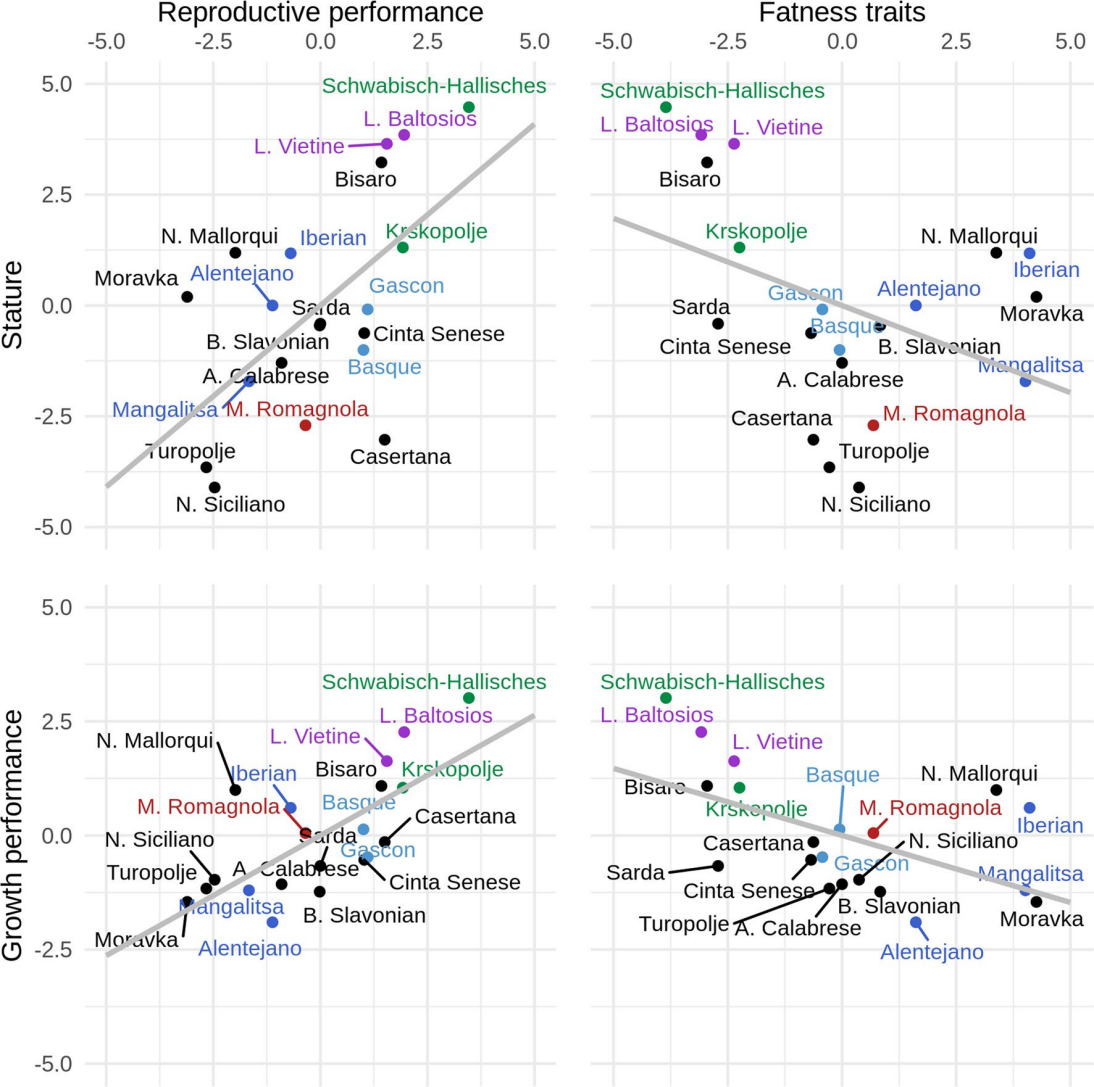
Global differences in production traits for 20 European local pig populations. Breeds are distributed according to breed scores for phenotypic traits and colored according to genetic similarity. Lower values for breed score growth represent lower average daily gain from weaning to 60 kg of live weight, while higher values represent higher average daily gain in the same growth period. Low values for breed score reproduction represent low reproductive performance, while high values represent breeds with higher reproductive performance. Low values for breed score stature represent lighter and smaller breeds, while high values represent heavier and larger breeds. Low values for the breed score fat represent leaner breeds, while high values represent fatter breeds. Breeds in green are genetically Landrace-like breeds, in purple Large White-like breeds, in blue Iberian-like breeds, in red Duroc-like breeds, and in light blue the Gascon and Basque breeds

Lastly, breed scores of the phenotypic trait groups were plotted to examine the global distribution of breeds and production traits in the local pig populations (Fig. 4). Figure 4 shows that breeds that are characterized by larger size and higher growth potential were also more reproductively efficient than smaller breeds that had a lower growth rate. In addition, fatter breeds were smaller and lighter and had a lower growth rate than leaner breeds that are larger.

### Detection of genomic regions associated with phenotypic differentiation

**Fig. 5:**
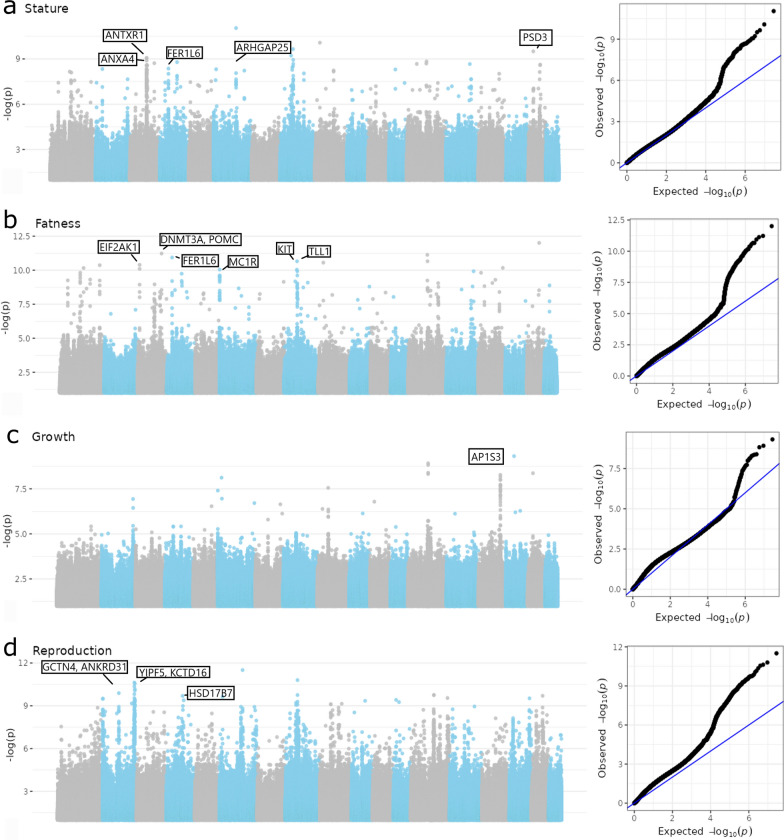
Manhattan plots for phenotypes associated with (**a**) stature, (**b**) fatness, (**c**) growth, and (**d**) reproduction and associated pp-plots

The approach proposed by Coop et al. [20] was extended to breed-level phenotypes to identify genomic regions potentially influencing phenotypic differentiation in local European pig breeds (“Methods” section). A scan of signatures of selection was performed for each phenotypic breed score, resulting in p-values for each SNP. Figure 5 shows Manhattan plots for stature, fatness, growth, and reproduction performance, along with a pp-plot that contrasts the expected and observed distributions of (−log10) p-values.

Across the genome, windows/regions with four significant SNPs at a significance threshold of 0.01 were considered as selection signals for phenotypes. Overall, 234 regions/windows were discovered, ranging in length from 3.1 kb to 1.282 Mb. Among the detected regions, 16 were in the stature group, two in the growth group, 24 in the fatness group, and 192 in the reproduction group. For a list of signatures of selection for these phenotypes, Additional file 5: Tables S5, S6.

Genomic regions identified for the stature phenotypic group ranged in length from 3.1 to 362.2 kb, with a maximum of 35 SNPs, and included from zero to four annotated genes. The region with the strongest signal included the *ARHGAP28* gene. The growth phenotypic group contained two regions with a maximum of 18 significant SNPs, which included one annotated gene (i.e. *AP1S3*). Genomic regions identified for fatness phenotypic group ranged in length from 3.1 to 590.5 kb, with a maximum of 30 SNPs, and included between 1 and up to 12 annotated genes. The region with the strongest signal included the *DNMT3A, POMC,* and *EFR3B* genes. Regions identified for the reproduction phenotypic group ranged in length from 12.8 to 1282 kb, with a maximum of 170 SNPs. The region with the strongest signals was located on *Sus scrofa* (SSC) chromosome SSC6 and contained the *YES1*, *ENOSF1*, *TYMS*, and *CLUL1* genes. A comprehensive view of the location of detected selection signals for production traits are shown on Additional file 2: Fig. S10.

**Fig. 6:**
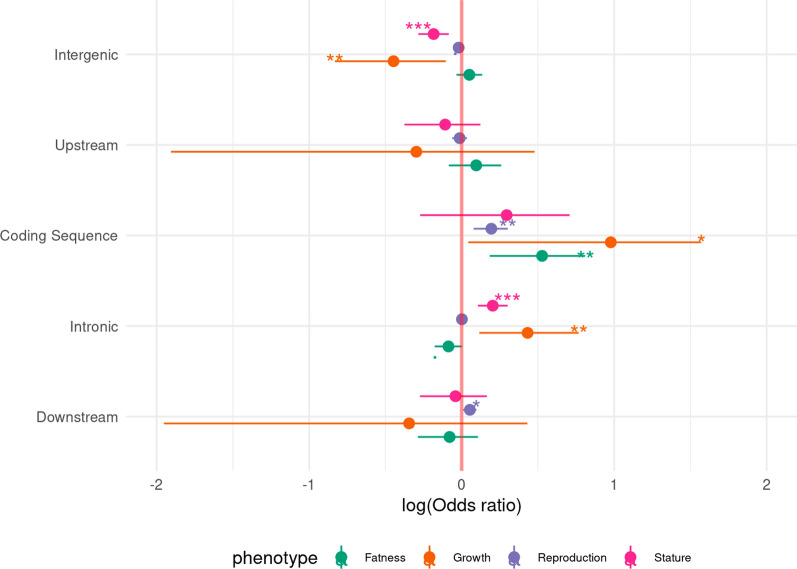
Enrichment of SNPs associated with different traits into functional categories

For each phenotypic group, we estimated the enrichment/depletion of significant SNPs in different functional categories, i.e. intergenic, upstream of genes, genic (coding regions or introns), and downstream of genes (Fig. 6). We observed a general trend for an enrichment of significant SNPs to be in genic regions (exons and introns) and some depletion in intergenic regions.

## Discussion

Deciphering the genetic basis of variation in complex traits can help understand their biology and evolution, and improve breeding, selection programs, and conservation plans for animal genetic resources. One approach to address this question is to link genetic to phenotypic variation by identifying genomic regions that have evolved in response to selection. Establishing such links requires a collection of data on genetic groups (e.g. populations, lines, breeds) that share a common origin and that have information on adaptive traits that were phenotyped in a standardized fashion. Livestock species generally meet these criteria and therefore provide good models for mapping genomic regions associated with selection. Here, we studied local pig populations that were genetically characterized using individual genotyping and DNA-pool sequencing and for which a common phenotypic database was recently established.

### Genetic diversity of European local pig breeds

Using individual genotyping data of a large set of European breeds, we found that their genetic variation is structured around four main genetic groups: Duroc, Large White, Landrace, and Iberian. The origin of the Duroc breed is unclear, as it originated from the American continent and was then brought back to Europe. Its history implies that it was separated from the European breeds for a long time, which is evident in our genetic analysis by its rather large genetic distance from most European breeds, except for the Mora Romagnola breed from Italy, which has a history of crossbreeding with the Duroc breed [33]. In addition, results from our PCA analyses show that the Mora Romagnola breed (and also the Turopolje breed) differentiated in the first components of the PCA analysis, which can be explained by their high degrees of inbreeding [34]. The White pig group is composed of the Lietuvos breed and the Large White breed, all of which originate from Northern Europe and are clearly separated from the Landrace breed, which includes populations that originated from more central European countries such as Germany (Landrace, Schwäbisch-Hällisches), Belgium (Pietrain), and Slovenia (Krškopolje). The Large White and Landrace groups are close to the root of the population tree, which can be explained by the well-documented influence of Asian pigs on these breeds [35]. The Iberian group includes a set of breeds that are quite geographically dispersed; while many of the Iberian-type breeds are found around southwestern Europe (Spain, Portugal, and France), some populations from Eastern/Central Europe clearly belong to the same genetic pool as the Iberian (e.g. Mangalitsa from Serbia, Turopolje from Croatia, and Cinta Senese from Italy). A possible interpretation is that the genetic background that is responsible for variation in the Iberian pig breed was historically widespread in Europe and can still be found in some local pig breeds. Another possibility to explain this result is recurrent admixture with wild boar [9, 36]. Overall, analysis of the genetic structure of European local pig breeds revealed extensive genetic variation that is clustered in differentiated breeds but with sometimes small genetic distances. The historical events that led to the present genetic variation among local breeds are most likely complex, involve differentiation from an ancestral pool followed or accompanied by outcrossing between populations, including wild boars, and cannot be reconstructed from the data used here. However, the sample of breeds used here is well adapted to our main objective, as we can interrogate a diverse set of polymorphisms for adaptation to contrasting environments or production systems in different genetic backgrounds.

While genotyping with a commercial SNP array is sufficient to characterize the genetic relationships and population structure of local pig breeds, the density of the array used here limits the resolution and power to detect potential associations with breed phenotypes, especially in local breeds, for which a large proportion of SNPs included on commercial arrays are not segregating. To alleviate this, a cost-effective alternative to SNP genotyping is DNA-pool sequencing of populations. In the dataset used here, we confirmed that allele frequency and genetic structure estimates obtained from DNA-pool sequencing are consistent with those obtained from a SNP array, while providing more comprehensive information on the genetic diversity of local pig breeds.

### Phenotypic diversity of European local pig breeds

Parallel to the comprehensive information on genetic diversity of local pig breeds, we developed an original approach based on PCA to characterize their phenotypic diversity for traits related to stature, growth, fatness, and reproduction, which have been described in a comprehensive analysis [3]. Local pig breeds have been raised under diverse management and production systems, which is reflected in their phenotypes. One example is the meat quality traits (pH24, pH48, and *longissimus dorsi* muscle color,), which are strongly influenced by pre-slaughter handling stress [37]. In addition, for these traits, many local breeds had a very high level of missing phenotyping data (> 50%) (Additional file 2: Fig. S11). Consequently, breed score for meat quality was not included in the phenotype-genotype selection scan.

Local pig breeds exhibit a wide variety of external phenotypic characteristics, including body size and body weight. This could be due to genetic differences or could reflect management of the breed. For example, males and females of the Schwäbisch-Hällisches breed are medium to large in size and are typically raised under well-developed management systems. In contrast, some of the less studied breeds (e.g. Moravka) are smaller and lighter [3] and managed in more heterogeneous systems.

Knowledge on the growth performance of local pig breeds is limited. Production systems may not be sufficiently adapted to the needs of local breeds, which could affect their growth. For example, Brossard et al. [38] argued that, animals are fed below ad libitum level in many studies on local pig breeds and, therefore, do not reach their full production potential. This is consistent with an analytical literature review [39] on European local pig breeds, which demonstrated that local pig breeds are generally fed ad libitum only earlier in life (during the growing and early fattening phase). In the late fattening phase, feeding is likely restricted. To limit the influence of such effects and to use a better representation of each breed’s growth potential, only average daily gain from the beginning of the lactation to the end of the early fattening phase (i.e. up to 60 kg) was considered for the phenotypic score for growth in our analyses. Nevertheless, observed breed differences in growth performance may still partly reflect differences in management, *e.g.* the Schwäbisch-Hällisches pig, which has a growth potential comparable to the genetically-improved modern pig breeds [40, 41].

It has been reported that local pig breeds have lower protein and higher lipid deposition capacities than modern pig breeds [38]. The higher capacity of local pig breeds for backfat and intramuscular fat deposition, and their lower muscle accretion have been demonstrated in several studies that have compared fat characteristics between local and modern breeds [42–46]. Although the local pig breeds are recognized as fatty, they still exhibited large breed differences for fatness traits in the data used here.

Regarding reproduction traits, sows of local breeds typically exhibit a relatively high age at first parturition, few litters per sow per year, long lactation periods, small litter sizes, and high piglet mortality [39]. Our PCA of reproductive performance distinguished breeds with better reproductive efficiency, which are usually reared in more intensive systems within more developed pork chains (e.g. Schwäbisch-Hällisches pig), from breeds with lower reproductive efficiency (e.g. Nero Siciliano, Turopolje, Casertana, Mora Romagnola, Mangalitsa), which are typically reared in extensive or semi-extensive systems [3].

While some of the observed breed differences in phenotypes can be explained by differences in production systems among local breeds, our genetic association results suggest that some of these differences may be genetic. In order to be able to test for such genetic associations, the methods used here require all breeds to have phenotypic information on the studied traits. To accomplish this, we imputed missing phenotypes of some breeds using PCA (see Methods). The resulting imputed phenotypes are likely to be biased towards the average of all breeds. This is for example most likely the case for the Sarda breed, which is considered to be a very small breed (in terms of body size) but had intermediate imputed size. This certainly limits power to detect genetic associations (false negatives) but will likely not create false positives.

### Genomic regions associated with phenotypic differentiation in European local pig breeds

To detect genomic regions associated with phenotypic differentiation of European pig populations, we built upon a method developed for genotype-by-environment associations [20], considering breed level phenotype as the environment. In our implementation of this approach, we tried to ensure robustness of our findings with respect to two main factors: (i) the imprecise estimate of allele frequencies from DNA-pool sequencing data and (ii) the heterogeneous nature of the phenotype data collected across breeds. First, we used regularized prediction of allele frequencies that ensures that loci with low informativeness (e.g. low sequencing depth) do not lead to extreme allele frequencies. Second, rather than testing associations with individual phenotypes, we used scores based on PCA that aggregate multiple phenotypes. This means that the influence of a single data point (breed, phenotype) on the phenotypic score analyzed is buffered. In spite of these two procedures, the empirical distribution of the p-values of the LRT statistic that was used as a measure of evidence for association was inflated towards low p-values. To address this, a permutation scheme to produce data under the null hypothesis (no association between phenotype and allele frequencies) was performed and used to obtain the final p-values that were used to identify significant genomic regions. It is important to point out that this approach is very different from scans of signatures of selection based on allele frequencies that detect either a local reduction in heterozygosity or an excess differentiation between populations and that do not use quantitative phenotype information. For example, on the one hand, selective sweeps not linked to the production phenotypes considered here can produce high signals of breed differentiation but no association with breed phenotype scores. On the other hand, more subtle shifts in allele frequencies due to phenotypic differentiation between breeds may be difficult to capture without testing for association with a specific phenotype. The degree of overlap for genomic regions identified with these two approaches is therefore difficult to predict beforehand because it depends on which factors affected the differentiation of breeds and on what phenotypes are available. Thus, the two approaches can be quite complementary, which must be taken into consideration when comparing the results of the two approaches using the same genetic data, as we have done using the findings of [8] (see below).

Turning now to the association results, the genome scans for selection associated with broad phenotypic trait groups revealed 234 regions associated with stature, fatness, growth, and reproduction traits. However, the number of regions identified differed substantially between trait groups, with 192 regions identified for the reproduction traits group but only two for the growth traits group. While the relative magnitude of these numbers could change slightly with the definition of a “significant region”, it is clear that the reproduction trait group exhibits more significant regions than the others. This could be the result of different, non-exclusive reasons. First, it seems that the permutation procedure to obtain p-values was less efficient for the reproduction trait scores than for the others, based on the slightly biased distribution of the p-values on the pp-plot of Fig. 5 for the reproduction trait analysis (Panel d on the right). Another possibility is that the reproduction traits are more influenced by the management systems than the other traits. As a result, testing for an association with the reproduction traits actually captures complex adaptations to other traits that were not recorded. The consequence is that using the reproductive score, we are actually capturing adaptation to many other traits that are associated with adaptation to the rearing conditions, resulting in a larger portion of the genome contributing to the response of local breeds to selection.

Relatively little overlap was found between the significant regions discovered with the trait-association approach here and the trait agnostic approach applied to the same DNA-pool sequencing data [8]. Only two regions were found to be clearly overlapping, one on SSC8, encompassing the *MAP9* gene but also close to the *KIT* gene, which is associated notably with coat color, and the other on SCC15, containing the *TMEM237* and *MPP4* genes. This illustrates the complementarity of the two approaches, and empirically highlights the gain in power that can be obtained by testing specific hypotheses (e.g. the association of allele frequencies with phenotype difference), as was shown in simulations studies [20, 21].

A strong association was found for stature and fatness in a region on SSC3 that contains the *ANXA4* gene. The *ANXA4* gene encodes a calcium-dependent phospholipid-binding protein that is involved in various membrane processes. This candidate gene and region was previously proposed as a quantitative trait locus (QTL) for stature in cattle [47, 48]. Another genomic region associated with a signature of selection for stature and growth was discovered on SCC3 and contained the candidate gene *ANTXR1*, which is involved in cell morphogenesis, cellular development process, and cytoskeleton organization. Part of this region was also previously proposed as a QTL for stature in cattle [49]. For the fatness and the stature trait groups, two overlapping regions were found on SCC3, which contain common candidate genes, including *DNMT3A* (responsible for CpG methylation), *POMC* (prohormone), and *EFR3B* (involved in localization of the phosphatidylinositol 4-kinase to the plasma membrane). The *DNMT3A* gene was previously shown to affect stature and body weight in cattle and humans [49–51] and also has a role in regulation of adipose tissue development [52]. Another candidate gene found in the same region is the *POMC* gene, which encodes the precursor of several peptide hormones that contribute to regulation of feed intake and energy balance via the leptin/melanocortin pathway [53]. Polymorphisms in the *POMC* gene have previously been associated with *longissimus dorsi* muscle area and backfat thickness in cattle [54–56] and with obesity and body mass index in humans [57, 58]. An interesting signature of selection associated with reproductive performance was found on SSC4, which contains the *HSD17B7* gene. This gene encodes an enzyme involved in the biosynthesis of sex steroids and cholesterol [59, 60]; and therefore is a good candidate gene for reproductive performance in local pig breeds.

Several other regions have also been identified as signatures of selection for stature and fatness but, although their connection to a biological function in production traits is not so direct, they do show some association with the phenotypes. These regions could be useful for further/future studies on signatures of selection. For example, SSC6 contained a region that might be associated with adaptation of stature and harbors the gene *ARHGAP28*, which was previously associated with the number of vertebrae in pigs, thus affecting carcass length [61]. In the fatness group, the gene *EIF2AK1* (located on SSC3) was identified, with a role in the inhibition of protein synthesis in response to stress. This gene was previously associated with body mass index in pigs [62].

Some of the regions that have been discovered here must be interpreted with care. For instance, the studies included in the meta-analysis differ in terms of production conditions. Therefore, correlations between phenotypic groups could create non-causal signals with genes. An example of such a non-causal signal found in the growth and fatness analyses is a region on SSC8 that contains the *KIT* gene. This gene encodes the tyrosine kinase receptor and is associated with coat color in pigs. Another signature of selection associated with coat color was found on SSC6 in the growth and fatness groups, which contains the *MC1R* gene that plays a major role in controlling the transition from eumelanin (black or brown) to pheomelanin (yellow to red) [63]. Since coat color is frequently part of breed standards, it was strongly selected for in several local breeds [64] (e.g. White breeds were under selection for leanness and better growth performance). Interestingly, a study performed on the same animals and breeds but using SNP-chip data [9] did not detect any signal near the *MC1R* or *KIT* genes, probably due to the different informativeness of the SNP-chip, as well as differences in the statistical approaches.

## Conclusions

In this study, we exploited several pig datasets to conduct a large meta-analysis of their genetic and phenotypic diversity. We presented how the two types of diversity can be linked with a statistical approach that builds on methods proposed to detect local adaptation. This led to the identification of genomic regions associated with breed divergence in production performance. The results exemplify how DNA-pool sequencing data associated with phenotype data at the breed level can offer a cost-effective alternative to individual-based approaches such as genome-wide association studies to identify and understand genetic factors associated with the evolution of quantitative traits.

### Supplementary Information

**Additional file 1: Table S1.** Summary of sequencing statistics (adapted from Bovo et al.[8]). The table represents the pooled whole-genome sequencing statistics. **Table S2.** Description of phenotypic traits used for phenotypic characterization of European local pig breeds. Description of the phenotypic traits included in the principal component analysis.

**Additional file 2: Figure S1.** Plot of admixture cross-validation error from K = 2 to 40. **Figure S2.** A matrix of missing phenotypic variables in a database of European local pig breeds. The yellow color represents missing variables. SFA = saturated fatty acid content, PUFA = polyunsaturated fatty acid content, Piglets W weight = piglets weaning weight, MUFA = monounsaturated fatty acid content, Litter W birth = litter weaning weight, LD IMF = *longissimus dorsi* intramuscular fat content, M = male, F = female, Death WN = death rate to weaning, BFT = backfat thickness, GM = *gluteus medius* muscle, ADG1 = Average daily gain during the lactation period, ADG2 = Average daily gain in the growing period from weaning to 30 kg of weight, ADG3 = Average daily gain during fattening period from 30 to 60 kg. **Figure S3.** Projection of the (A) stature, (B) growth, (C) fatness and (D) reproduction imputed data obtained with iterative principal component algorithm. **Figure S4.** Admixture plots of European local pig breeds. The circular plot represents admixture plots for K = 24, K = 20, K = 15, K = 10, and K = 6 (from the outside to the inside of the circle). **Figure S5.** Comparison of SNP array and DNA-pool sequencing data. Left: allele frequencies estimated from SNP array genotypes vs DNA-pool sequencing data for each local breed. R are the correlation coefficients (all p-values < 1e-16). Right: projection of the DNA-pool sequencing data (allele frequencies) on the PCA established with individual genotypes on the SNP genotyping array. The x-axis is the average coordinate of individuals of each population on each of the PC of the genotyped-based PCA, and the y-axis is the coordinate of the allele frequencies of each population projected on each PCA. **Figure S6.** Principal component analysis showing the relationship between breeds (A) and the traits associated with growth (B) and the corresponding phenotypic breed scores (C). Breeds (A) colored in grey are the breeds with more than 50% of missing variables, thus, their position on the PCA must be interpreted with care. The variables (B) are colored according to quality of the representation, which is measured by squared cosine between the vector originating from the element and its projection on the axis. The variables that contribute most to the separation of the trait into PC1 and PC2 are colored black. Breeds (A, C) are colored according to genetic similarity. Breeds (A, C) in green are genetically Landrace-like breeds, in purple are Large White-like breeds, in blue are Iberian-like breeds, in red are Duroc-like breeds and in light blue are Gascon and Basque. **Figure S7.** Principal component analysis showing the relationship between breeds (A) and the traits associated with fatness (B) and the corresponding phenotypic breed scores (C). Breeds (A) colored in grey are the breeds with more than 50% of missing variables, thus, their position on the PCA must be interpreted with care. The variables (B) are colored according to quality of the representation, which is measured by squared cosine between the vector originating from the element and its projection on the axis. The variables that contribute most to the separation of the trait into PC1 and PC2 are colored black. Breeds (A, C) are colored according to genetic similarity. Breeds (A, C) in green are genetically Landrace-like breeds, in purple are Large White-like breeds, in blue are Iberian-like breeds, in red are Duroc-like breeds and in light blue are Gascon and Basque. **Figure S8.** Principal component analysis showing the relationship between breeds (A) and the traits associated with reproduction performance (B) and the corresponding phenotypic breed scores (C). Breeds (A) colored in grey are the breeds with more than 50% of missing variables, thus, their position on the PCA must be interpreted with care. The variables (B) are colored according to quality of the representation, which is measured by squared cosine between the vector originating from the element and its projection on the axis. The variables that contribute most to the separation of the trait into PC1 and PC2 are colored black. Breeds (A, C) are colored according to genetic similarity. Breeds (A, C) in green are genetically Landrace-like breeds, in purple are Large White-like breeds, in blue are Iberian-like breeds, in red are Duroc-like breeds and in light blue are Gascon and Basque. **Figure S9.** Distribution of the European local pig breeds according to intramuscular fat content in *longissimus dorsi* (LD) muscle. Higher values on x-axis represent higher intramuscular fat content, while the lower values represent lower intramuscular fat content. **Figure S10.** Circular plot summarizing regions with candidate genes for phenotypic groups growth, stature, fatness and reproduction. Discovered regions associated with growth are colored in red, with stature are colored in blue, with fatness are colored in purple and with reproductive performance in green. **Figure S11.** Principal component analysis showing the relationship between breeds (A) and the traits associated with meat quality (B) and the corresponding phenotypic breed scores (C). Breeds (A) colored in grey are the breeds with more than 50% of missing variables, thus, their position on the PCA must be interpreted with care. The variables (B) are colored according to quality of the representation, which is measured by squared cosine between the vector originating from the element and its projection on the axis. The variables that contribute most to the separation of the trait into PC1 and PC2 are colored black. Breeds (A, C) are colored according to genetic similarity. Breeds (A, C) in green are genetically Landrace-like breeds, in purple are Large White-like breeds, in blue are Iberian-like breeds, in red are Duroc-like breeds and in light blue are Gascon and Basque.

**Additional file 3: Table S3.** Mean values for phenotypic traits and information on the number of considered experimental units and animals. **Table S4.** Mean values for breed scores

**Additional file 4: Figure S12.** The genetic structure of European local pig populations assessed from individual SNP genotyping data using principal component analysis.

**Additional file 5: Table S5.** A list of detected genomic regions associated with phenotypic differentiation in production traits in European local pig breeds. **Table S6.** List of detected genomic regions associated with phenotypic differentiation in reproduction traits in European local pig breeds.

## Data Availability

Data used in this study are available from the original sources as detailed in the Methods section.
