# Supplementary material for: A meta-analysis of genetic and phenotypic diversity of European local pig breeds reveals genomic regions associated with breed differentiation for production traits
Source: Genet Sel Evol. 2023 Dec 7;55:88. doi: 10.1186/s12711-023-00858-3 (PMC10704730; doi:10.1186/s12711-023-00858-3)
Supplement: Supplementary file 1 — Additional file 1: Table S1. Summary of sequencing statistics (adapted from Bovo et al.[8]). The table represents the pooled whole-genome sequencing statistics. Table S2. Description of phenotypic traits used for phenotypic characterization of European local pig breeds. Description of the phenotypic traits included in the principal component analysis. [file 12711_2023_858_MOESM1_ESM.pdf]

Additional information 1

A meta-analysis of the genetic and phenotypic diversity of European local pig breeds reveals genomic regions associated with their differentiation for production traits

Klavdija Poklukar, Camille Mestre, Martin Škrlep, Marjeta Čandek-Potokar, Cristina Ovilo, Luca Fontanesi, Juliette Riquet, Samuele Bovo, Giuseppina Schiavo, Anisa Ribani, Maria Muñoz, Maurizio Gallo, Ricardo Bozzi, Rui Charneca, Raquel Quintanilla, Goran Kušec, Marie-José Mercat, Christoph Zimmer, Violeta Razmaite, Jose P. Araujo, Čedomir Radović, Radomir Savić, Danijel Karolyi, and Bertrand Servin

Additional File 1: Table S1

Additional File 1: Table S2

Table S1. Summary of sequencing statistics (adapted from Bovo et. al [8]).

Population	No. of read pairs	Depth of coverage *
Alentejano	419,690,476	41.98
Apulo-Calabrese	418,529,727	42.12
Basque	407,698,128	39.55
Bísaro	415,284,437	42.44
Black Slavonian	405,316,112	40.61
Casertana	435,598,516	43.61
Cinta Senese	422,120,850	42.42
Gascon	408,764,207	41.10
Krškopolje	404,204,144	40.80
Lietuvus Vietiné	409,935,460	41.99
Lietuvus Baltosios Senojo Tipo	405,822,217	41.62
Negre Mallorquí	414,314,159	41.92
Mora Romagnola	411,095,541	41.21
Moravka	413,100,992	42.27
Nero Siciliano	405,812,223	38.92
Sarda	442,035,147	44.32
Schwäbisch-Hällisches	428,982,876	42.69
Mangalitsa	416,663,891	41.08
Turopolje	416,663,891	42.61
Italian Duroc	420,384,723	41.91
Italian Landrace	442,780,637	44.35
Italian Large White	450,673,024	45.24

* after removal of duplicated reads

Table S2. Description of phenotypic traits used for phenotypic characterisation of European local pig breeds.

Group	Trait code	Description of the trait
Growth performance	ADG 1	Average daily gain during the lactation period.
	ADG 2	Growing period from weaning to 30 kg of weight.
	ADG 3	First fattening period from 30 to 60 kg,
Stature	Bodyweight - M	Average male body weight.
	Bodyweight - F	Average female body weight.
	Height at withers - M	Male height at withers.
	Height at withers -F	Female height at withers.
Fatness	BFT last rib	Backfat thickness at the level of the last rib.
	BFT withers	Backfat thickness on withers.
	BFT at GM	Backfat thickness above *gluteus medius*.
	LD IMF	Intramuscular fat content in *longissimus dorsi* muscle.
	Meatiness	Meat content.
	Loin eye area	Loin eye area.
	SFA	Saturated fatty acid content of *longissimus dorsi* muscle.
	MUFA	Monounsaturated fatty acid content of *longissimus dorsi* muscle.
	PUFA	Polyunsaturated fatty acid content of *longissimus dorsi* muscle.
	BFT last rib LM	Adjusted values for backfat thickness at the level of the last rib on final body weight of 120 kg using linear mixed models.
Reproductive performance	Sow age	Age of sow in months at first parturition.
	Litter/year	Litters per sow per year.
	L W birth	Litter weight at birth in kg.
	Piglets/litter	Piglets per litter.
	Alive piglets/litter	Piglets alive per litter.
	Piglet live BW	Piglet live birth weight in kg.
	Stillborn/litter	Stillborn pigs per litter.
	Death WN	Death rate to weaning.
	Weaned piglets/litter	Piglets weaned per litter.
	Piglets W weight	Piglets weaning weight.
	Lactation	Duration of lactation.
	Farrowing interval	Farrowing interval in days.
