# Supplementary material for: A meta-analysis of genetic and phenotypic diversity of European local pig breeds reveals genomic regions associated with breed differentiation for production traits
Source: Genet Sel Evol. 2023 Dec 7;55:88. doi: 10.1186/s12711-023-00858-3 (PMC10704730; doi:10.1186/s12711-023-00858-3)
Supplement: Supplementary file 4 — Additional file 4: Figure S12. The genetic structure of European local pig populations assessed from individual SNP genotyping data using principal component analysis. [file 12711_2023_858_MOESM4_ESM.pdf]

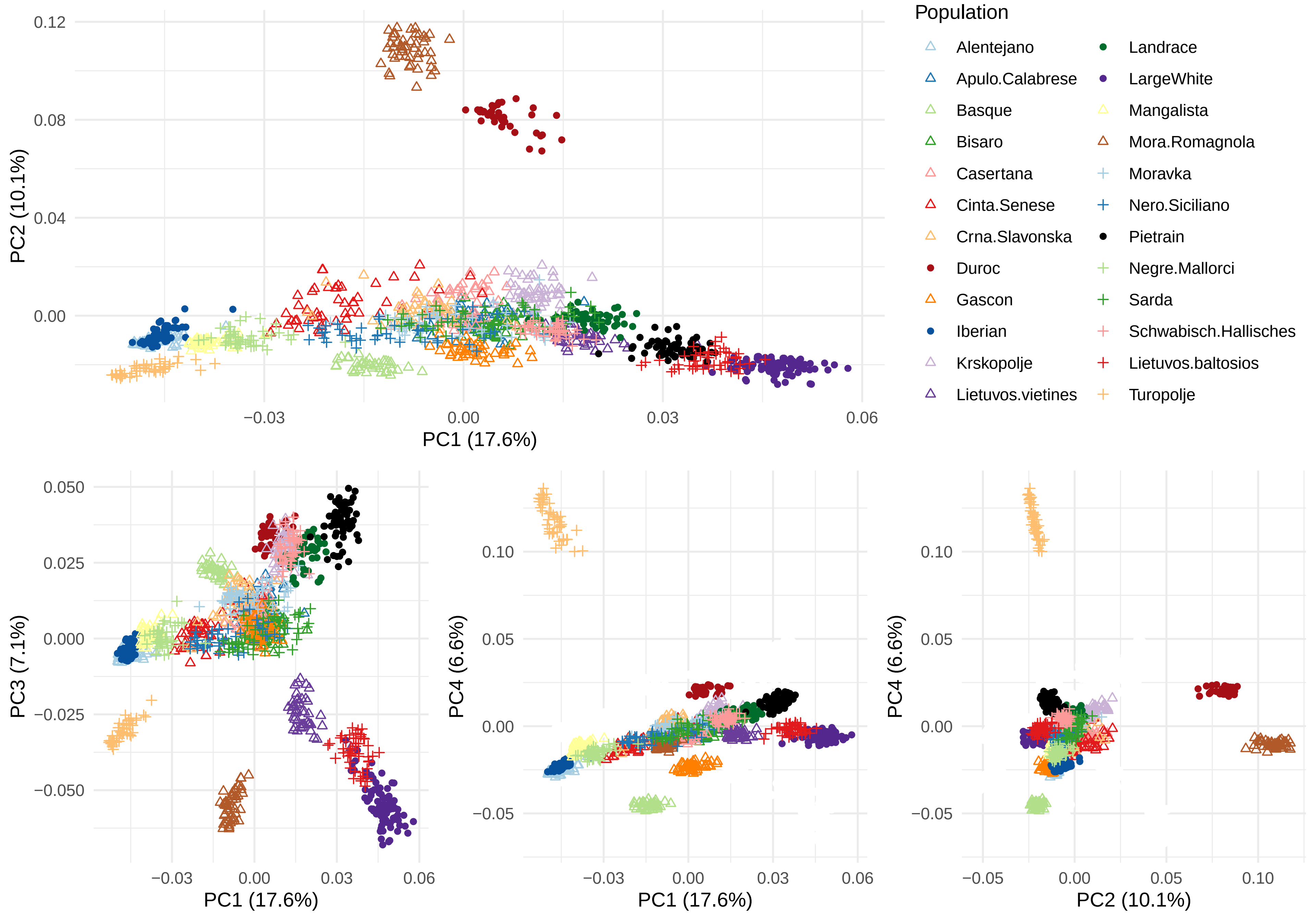
Population
Alentejano
Apulo.Calabrese
Basque
Bisaro
Casertana
Cinta.Senese
Crna.Slavonska
Duroc
Gascon
Iberian
Krskopolje
Lietuvos.vietines
Landrace
LargeWhite
Mangalista
Mora.Romagnola
Moravka
Nero.Siciliano
Pietrain
Negre.Mallorci
Sarda
Schwabisch.Hallisches
Lietuvos.baltosios
Turopolje
PC1 (17.6%)
PC2 (10.1%)
PC3 (7.1%)
PC4 (6.6%)
0.12
0.08
0.04
0.00
−0.03
0.03
0.06
0.050
0.025
0.000
−0.025
−0.050
0.10
0.05
−0.05

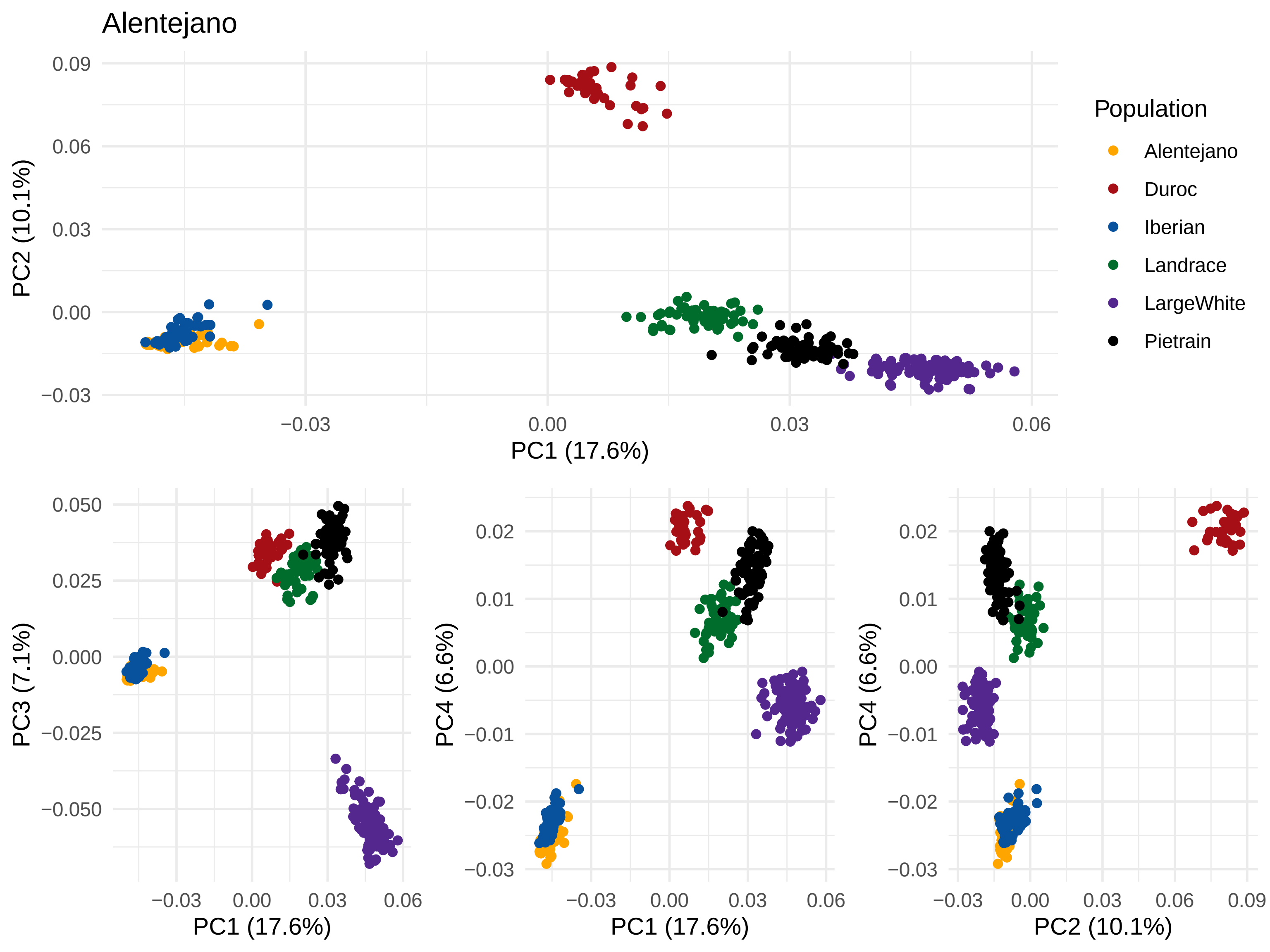
Alentejano
PC2 (10.1%)
PC1 (17.6%)
0.09
0.06
0.03
0.00
−0.03
−0.03
0.00
0.03
0.06
Population
Alentejano
Duroc
Iberian
Landrace
LargeWhite
Pietrain
PC3 (7.1%)
0.050
0.025
0.000
−0.025
−0.050
PC4 (6.6%)
0.02
0.01
0.00
−0.01
−0.02
−0.03
PC2 (10.1%)
0.09

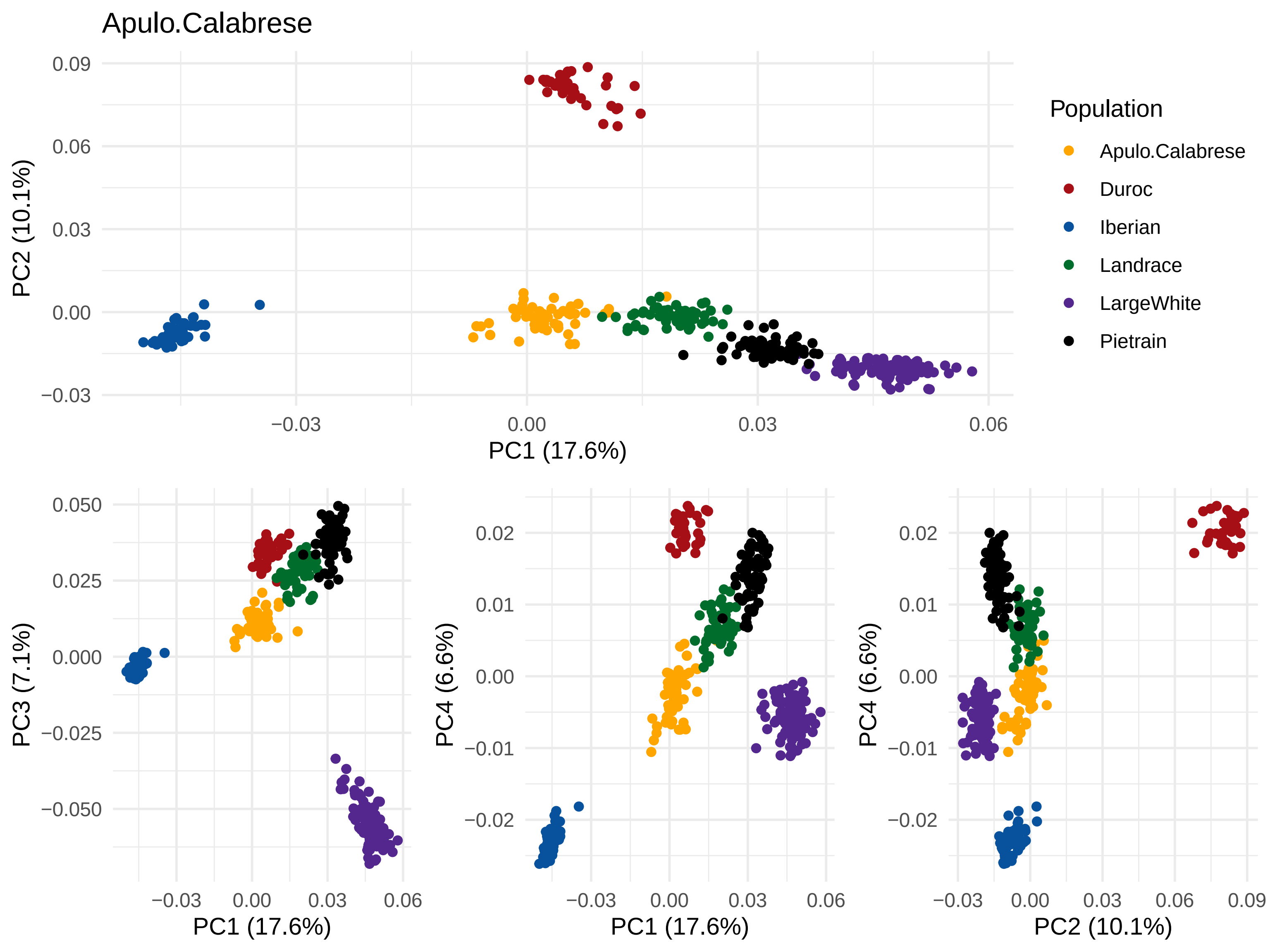

Apulo.Calabrese
PC1 (17.6%)
PC2 (10.1%)
PC3 (7.1%)
PC4 (6.6%)
Population
Apulo.Calabrese
Duroc
Iberian
Landrace
LargeWhite
Pietrain

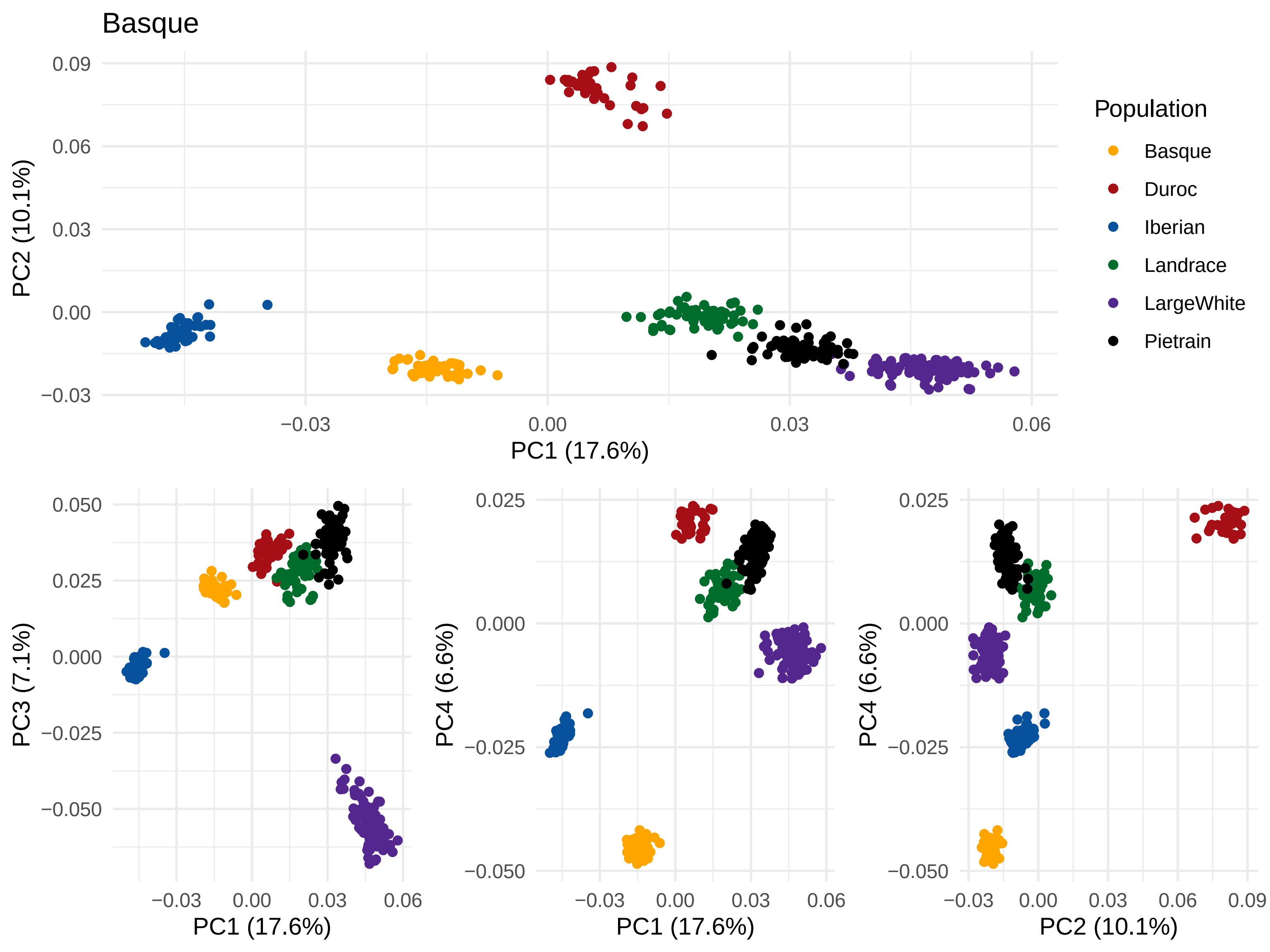

Basque
Population
Basque
Duroc
Iberian
Landrace
LargeWhite
Pietrain
PC1 (17.6%)
PC2 (10.1%)
PC3 (7.1%)
PC4 (6.6%)
0.09
0.06
0.03
0.00
−0.03
0.050
0.025
0.000
−0.025
−0.050

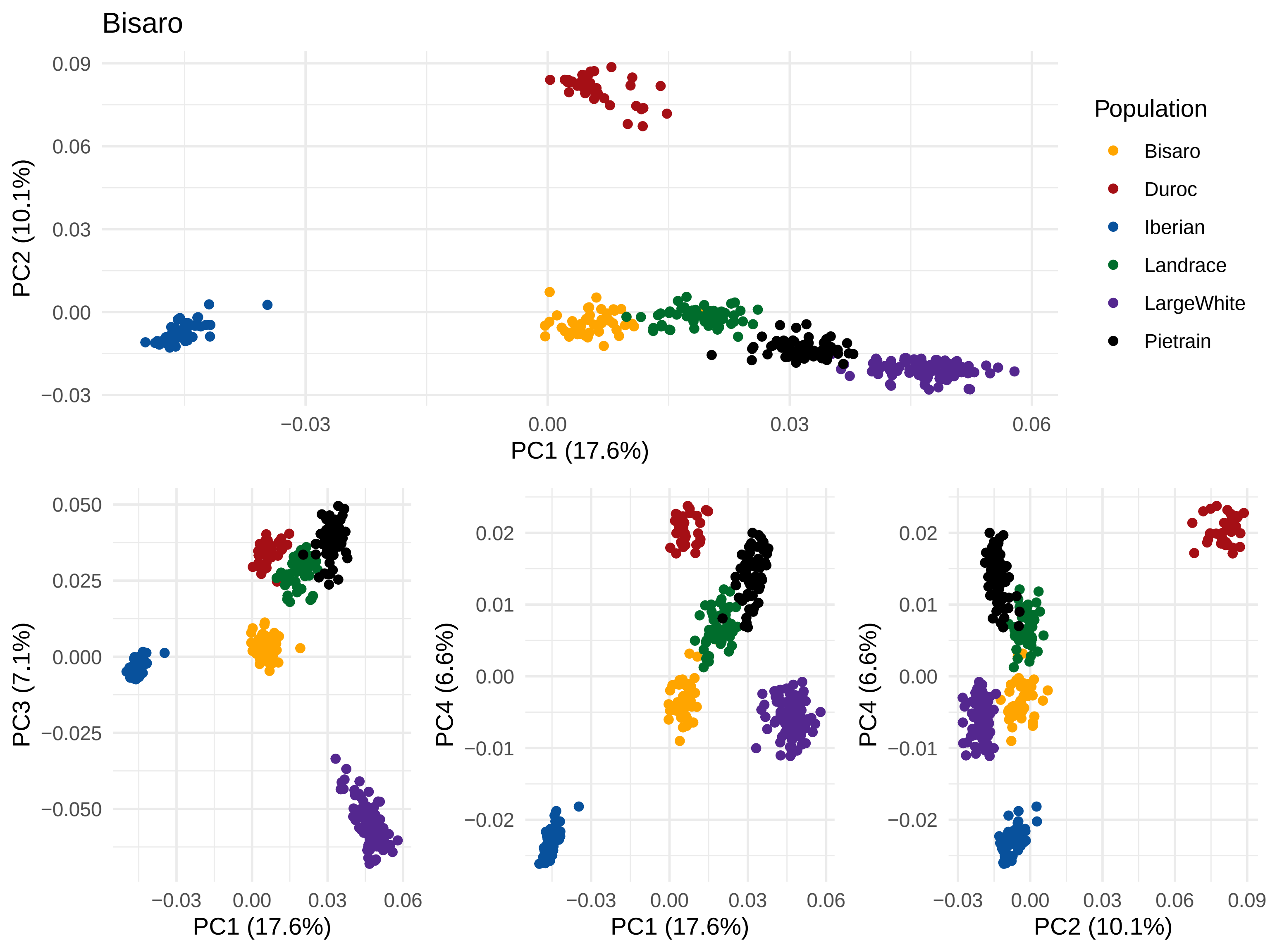
Bisaro
PC2 (10.1%)
PC1 (17.6%)
Population
Bisaro
Duroc
Iberian
Landrace
LargeWhite
Pietrain
0.09
0.06
0.03
0.00
−0.03
0.06
PC3 (7.1%)
0.050
0.025
0.000
−0.025
−0.050
PC4 (6.6%)
0.02
0.01
−0.01
−0.02
PC2 (10.1%)

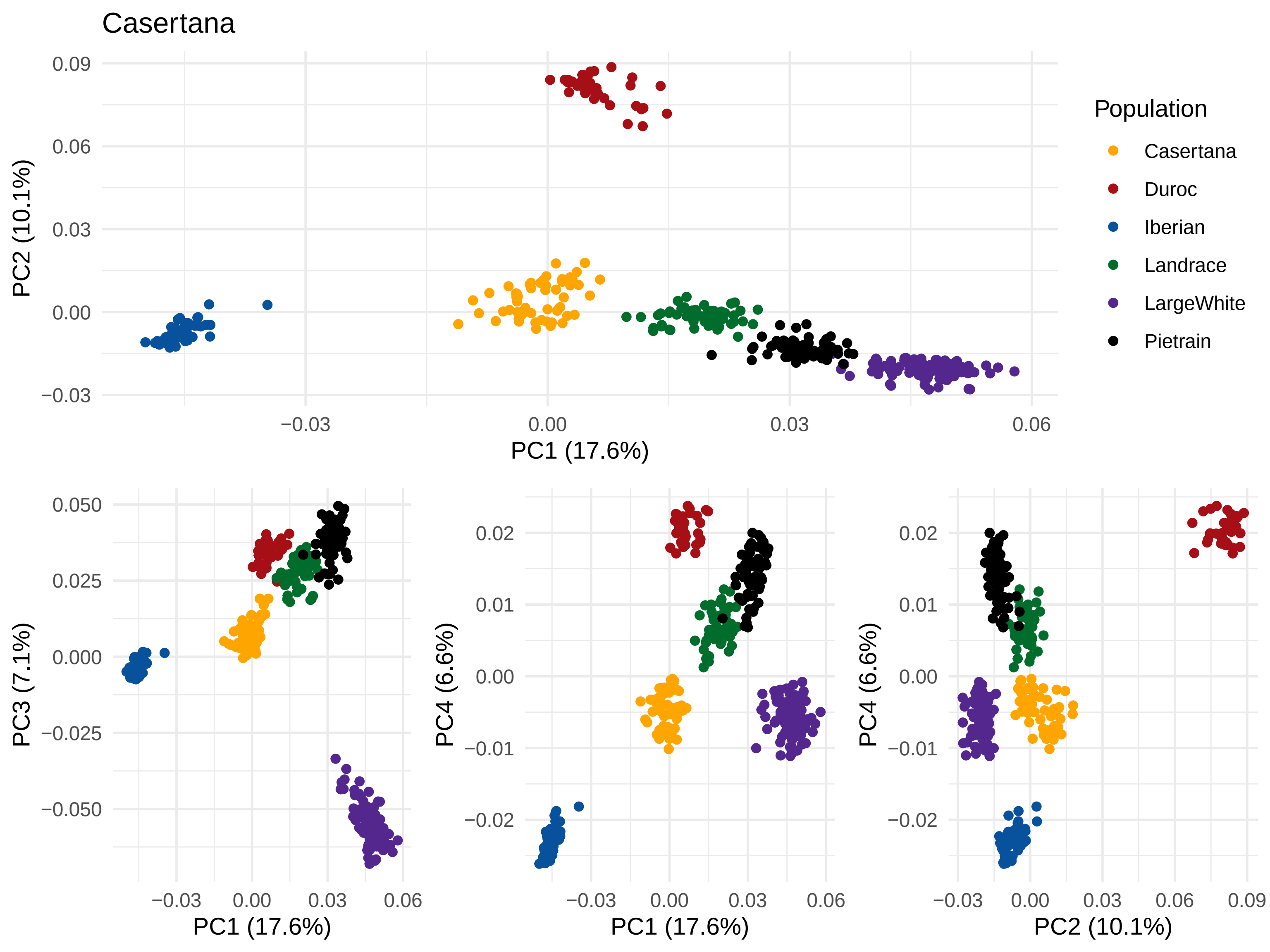

Casertana
0.09
0.06
0.03
0.00
−0.03
PC2 (10.1%)
−0.03
0.00
0.03
0.06
PC1 (17.6%)
Population
Casertana
Duroc
Iberian
Landrace
LargeWhite
Pietrain
0.050
0.025
0.000
−0.025
−0.050
PC3 (7.1%)
PC1 (17.6%)
0.02
0.01
0.00
−0.01
−0.02
PC4 (6.6%)
PC1 (17.6%)
PC4 (6.6%)
−0.03
0.00
0.03
0.06
0.09
PC2 (10.1%)

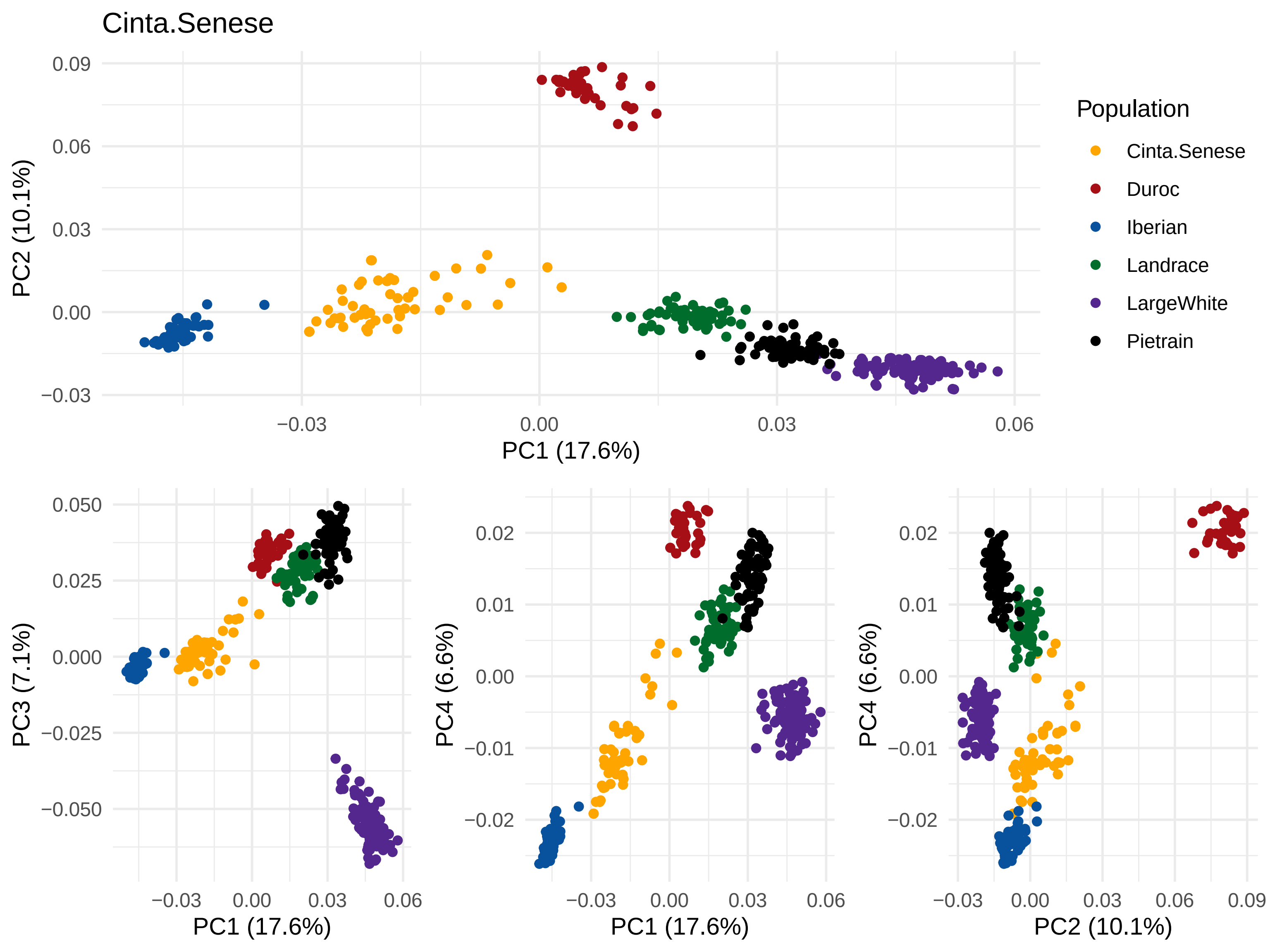

Cinta.Senese
PC1 (17.6%)
PC2 (10.1%)
PC3 (7.1%)
PC4 (6.6%)
Population
Cinta.Senese
Duroc
Iberian
Landrace
LargeWhite
Pietrain

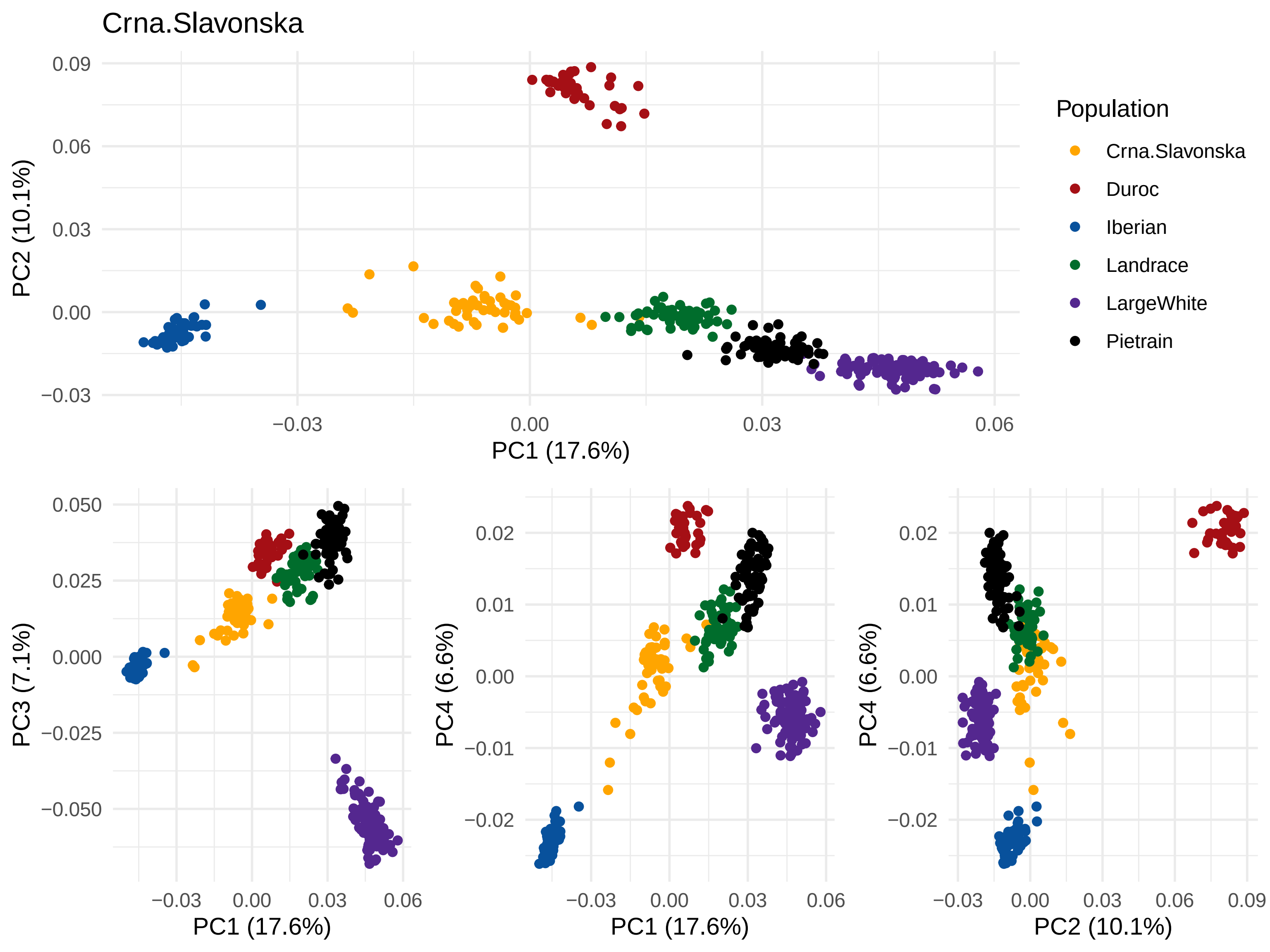

Crna.Slavonska
PC1 (17.6%)
PC2 (10.1%)
PC3 (7.1%)
PC4 (6.6%)
Population
Crna.Slavonska
Duroc
Iberian
Landrace
LargeWhite
Pietrain

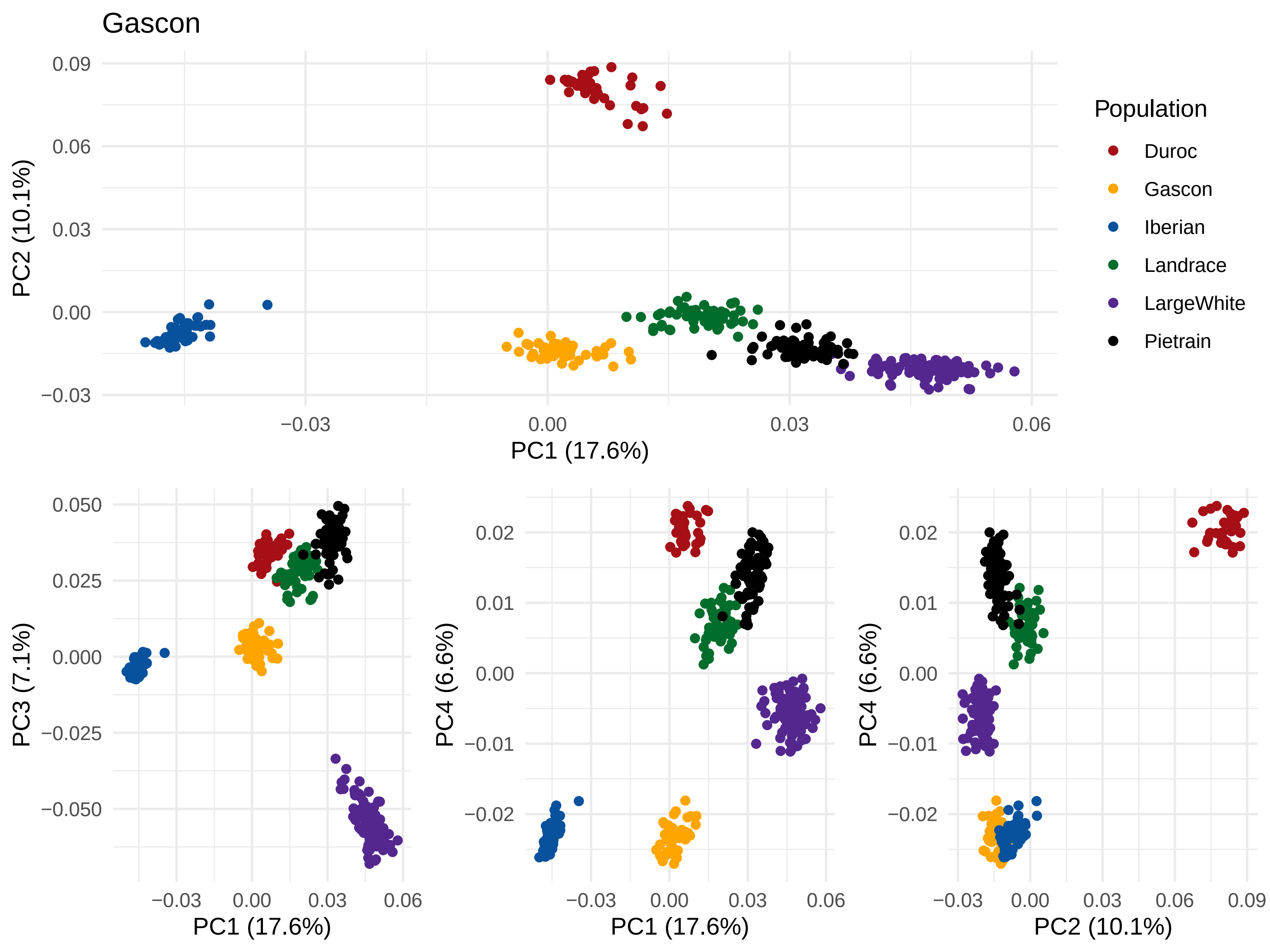
Gascon
PC2 (10.1%)
PC1 (17.6%)
Population
Duroc
Gascon
Iberian
Landrace
LargeWhite
Pietrain
PC3 (7.1%)
PC4 (6.6%)
0.09
0.06
0.03
0.00
−0.03
0.050
0.025
0.000
−0.025
−0.050
0.02
0.01
−0.01
−0.02

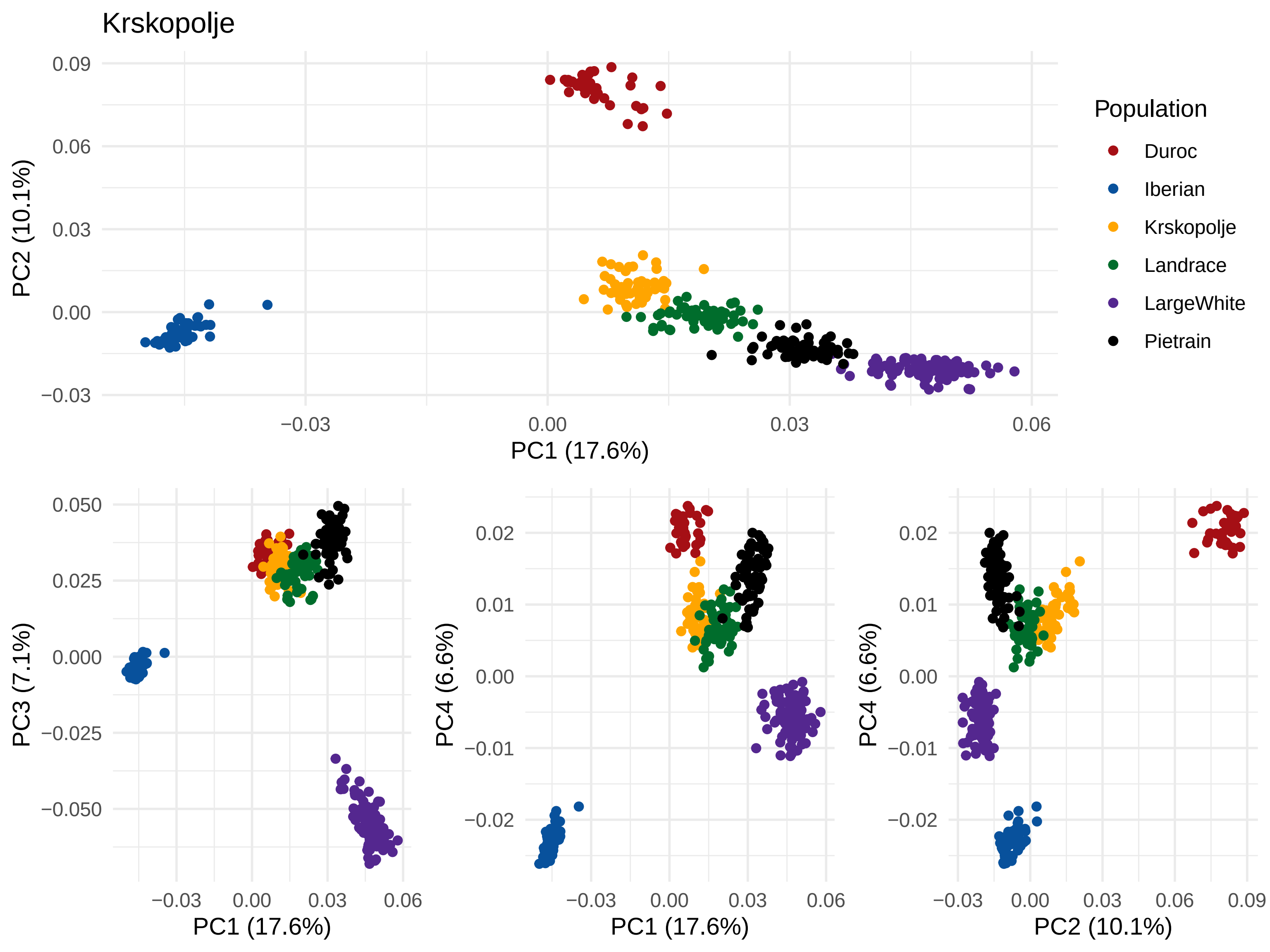

Krskopolje
PC1 (17.6%)
PC2 (10.1%)
PC3 (7.1%)
PC4 (6.6%)
Population
Duroc
Iberian
Krskopolje
Landrace
LargeWhite
Pietrain

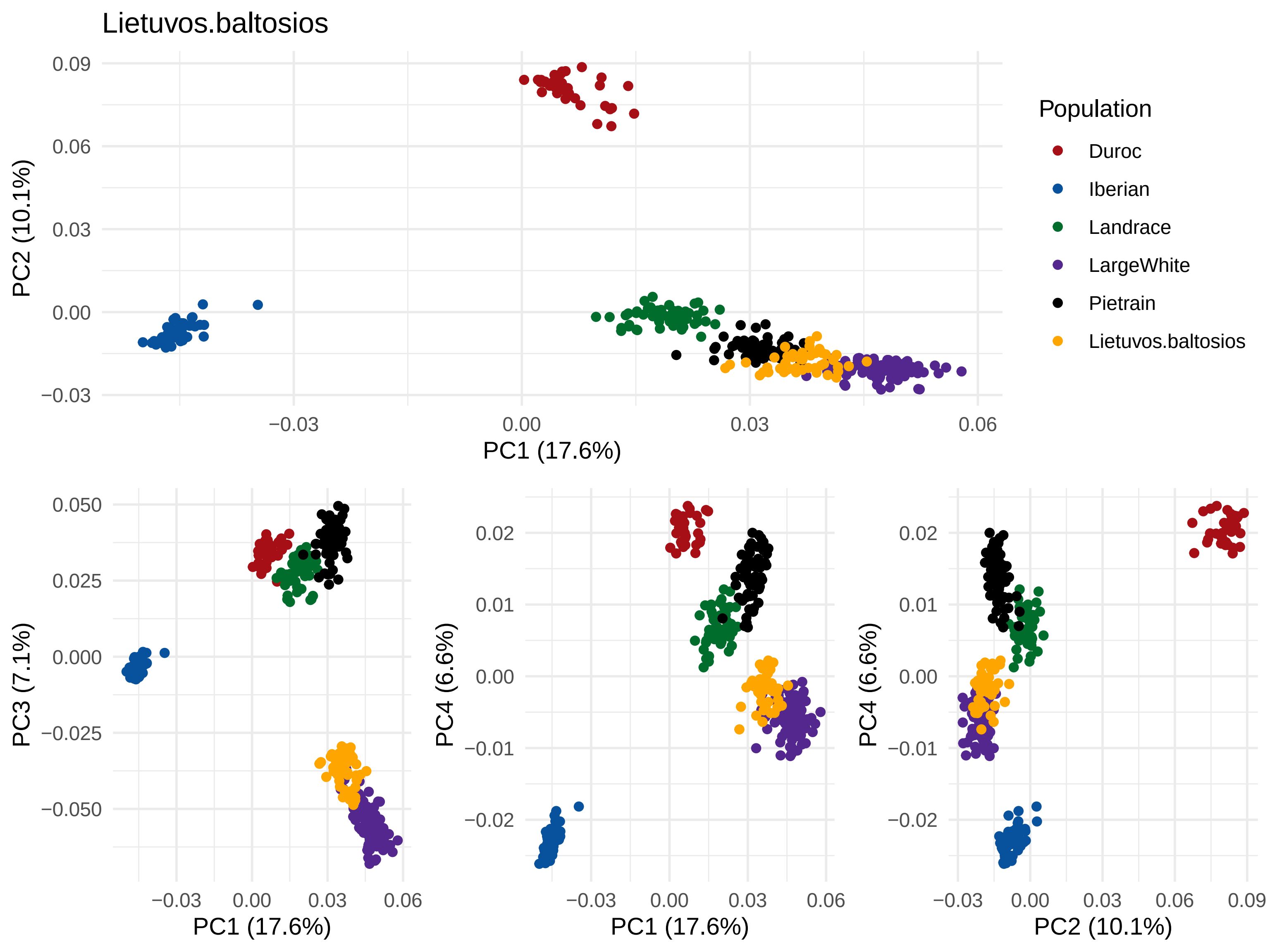

Lietuvos.baltosios
PC2 (10.1%)
PC1 (17.6%)
Population
Duroc
Iberian
Landrace
LargeWhite
Pietrain
Lietuvos.baltosios
0.09
0.06
0.03
0.00
−0.03
0.00
0.03
0.06
PC3 (7.1%)
0.050
0.025
0.000
−0.025
−0.050
PC4 (6.6%)
0.02
0.01
0.00
−0.01
−0.02
PC1 (17.6%)
PC2 (10.1%)

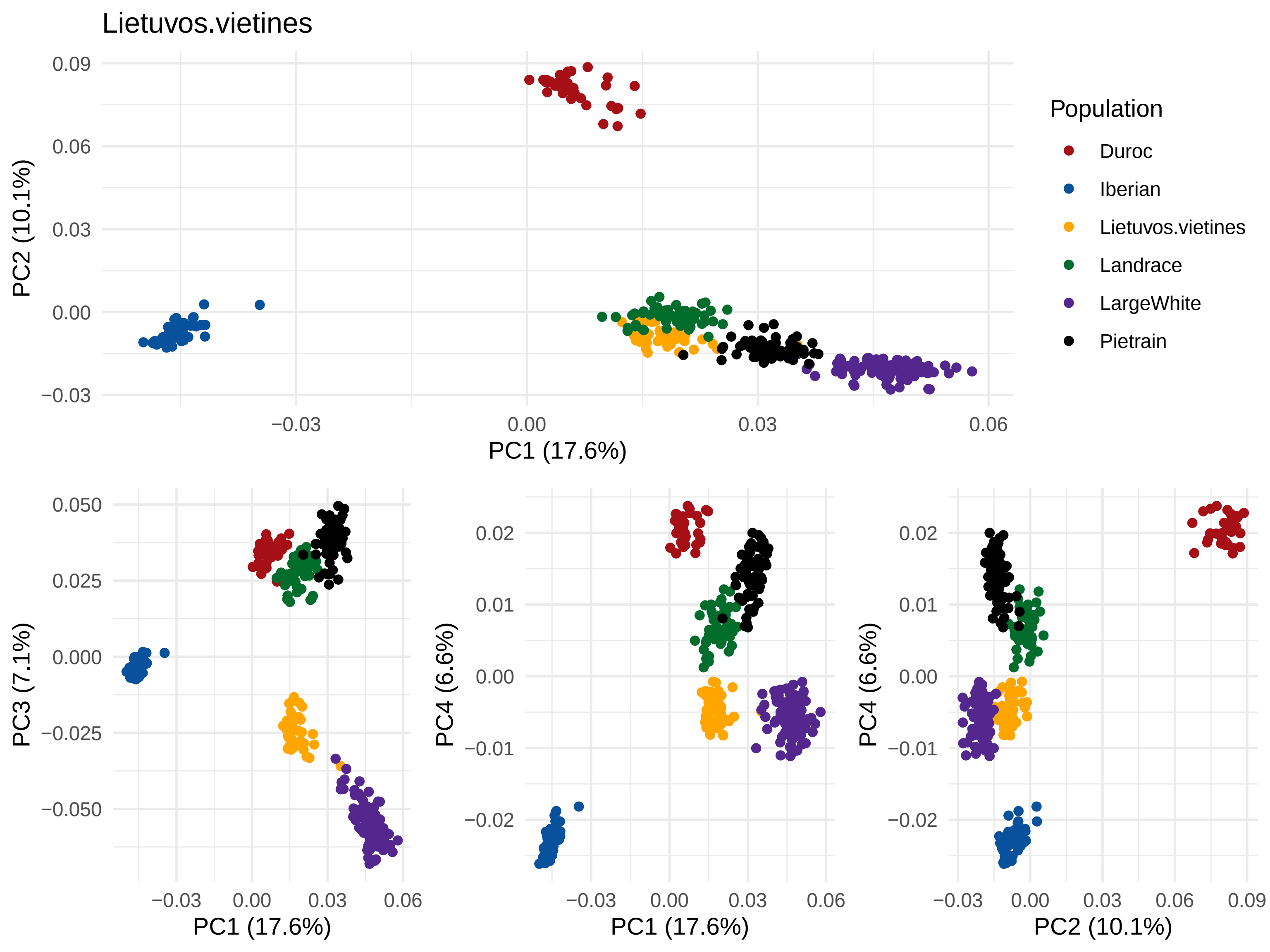
Lietuvos.vietines
PC2 (10.1%)
PC1 (17.6%)
Population
Duroc
Iberian
Lietuvos.vietines
Landrace
LargeWhite
Pietrain
PC3 (7.1%)
PC1 (17.6%)
PC4 (6.6%)
PC1 (17.6%)
PC4 (6.6%)
PC2 (10.1%)

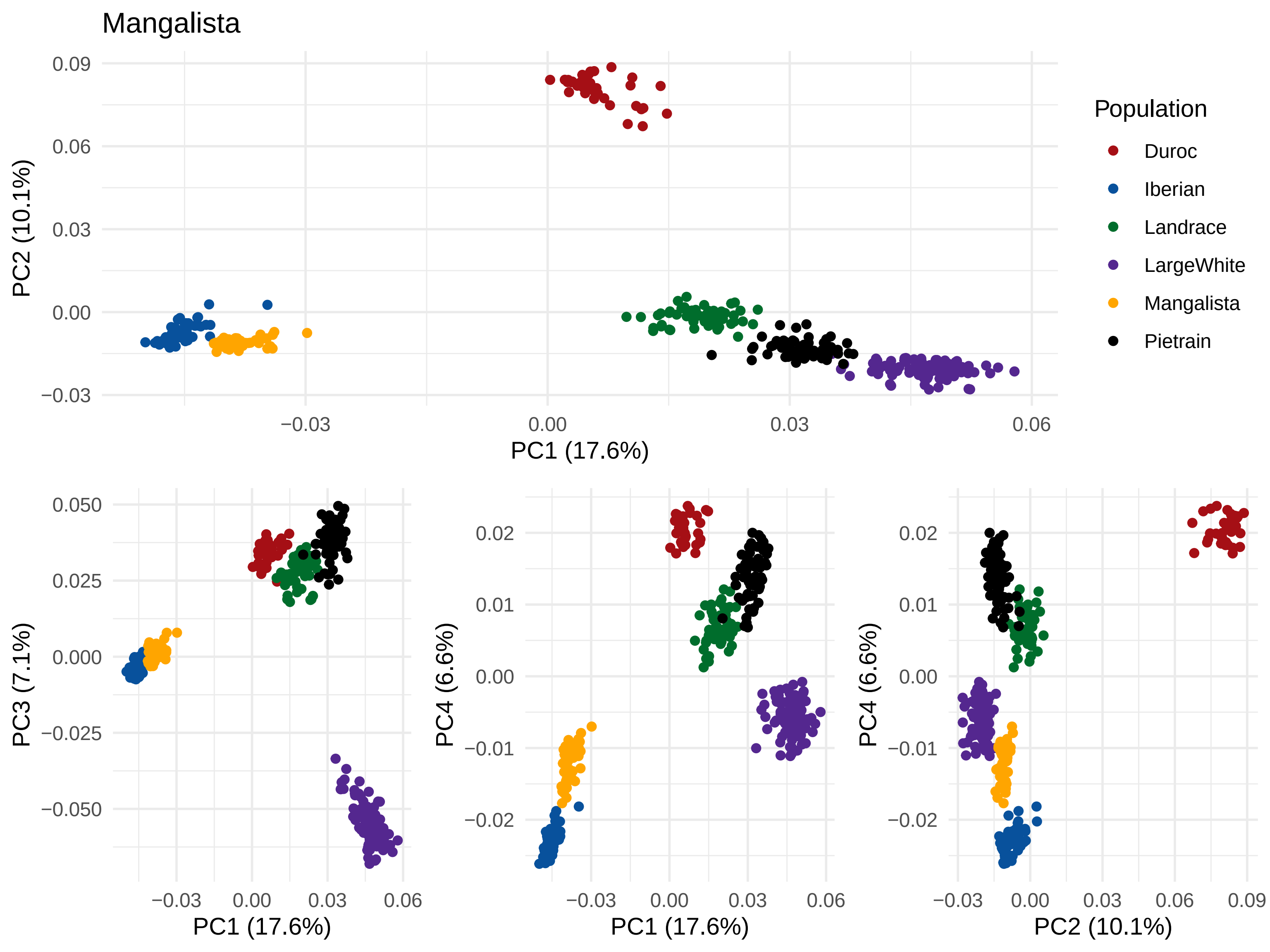

Mangalista
PC2 (10.1%)
PC1 (17.6%)
Population
Duroc
Iberian
Landrace
LargeWhite
Mangalista
Pietrain
0.09
0.06
0.03
0.00
−0.03
−0.03
0.00
0.03
0.06
PC3 (7.1%)
0.050
0.025
0.000
−0.025
−0.050
PC4 (6.6%)
0.02
0.01
0.00
−0.01
−0.02
PC2 (10.1%)

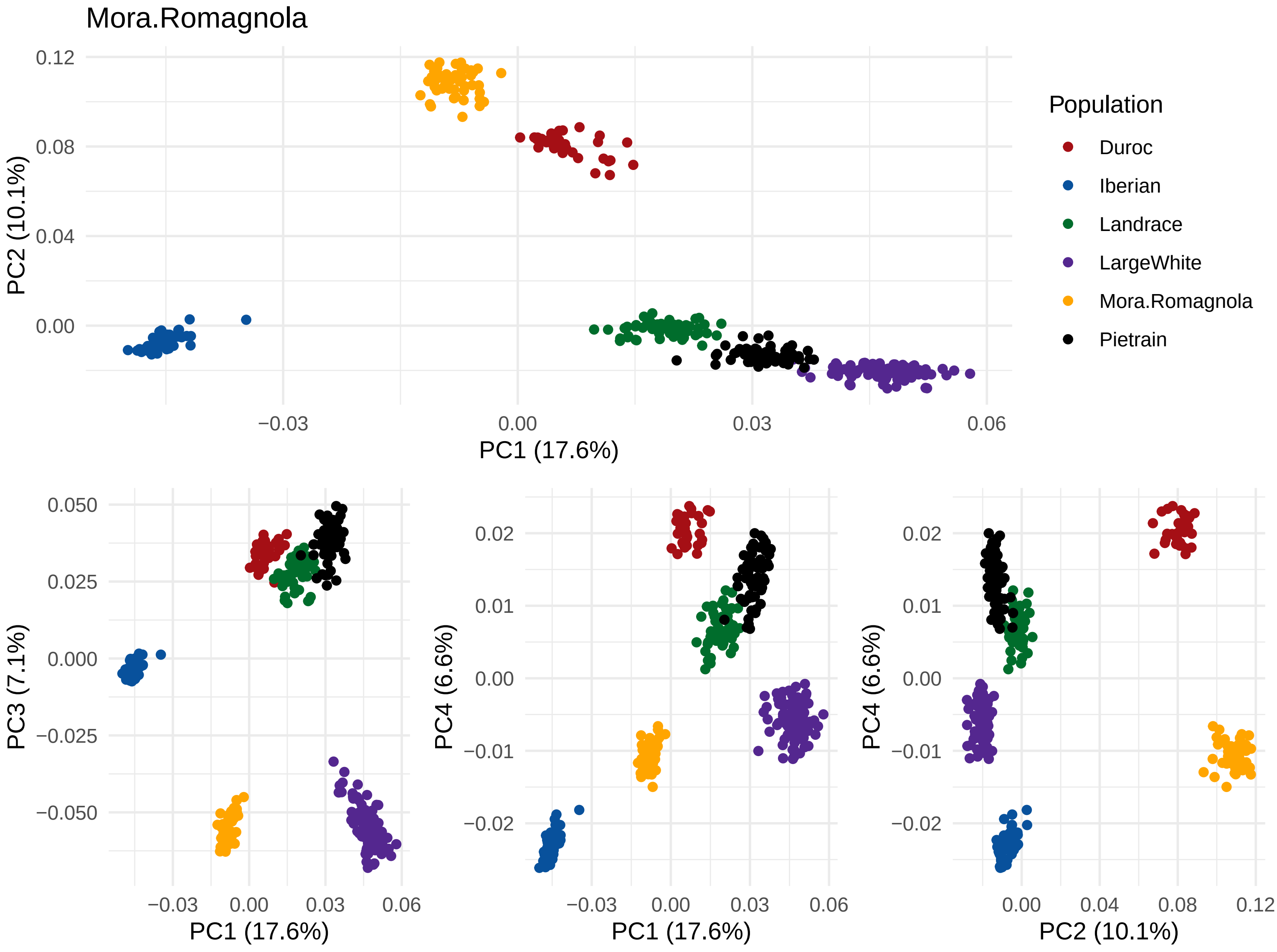
Mora.Romagnola
Population
Duroc
Iberian
Landrace
LargeWhite
Mora.Romagnola
Pietrain
PC2 (10.1%)
PC1 (17.6%)
PC3 (7.1%)
PC4 (6.6%)
0.12
0.08
0.04
0.00
−0.03
0.03
0.06
0.050
0.025
0.000
−0.025
−0.050
0.02
0.01
−0.01
−0.02

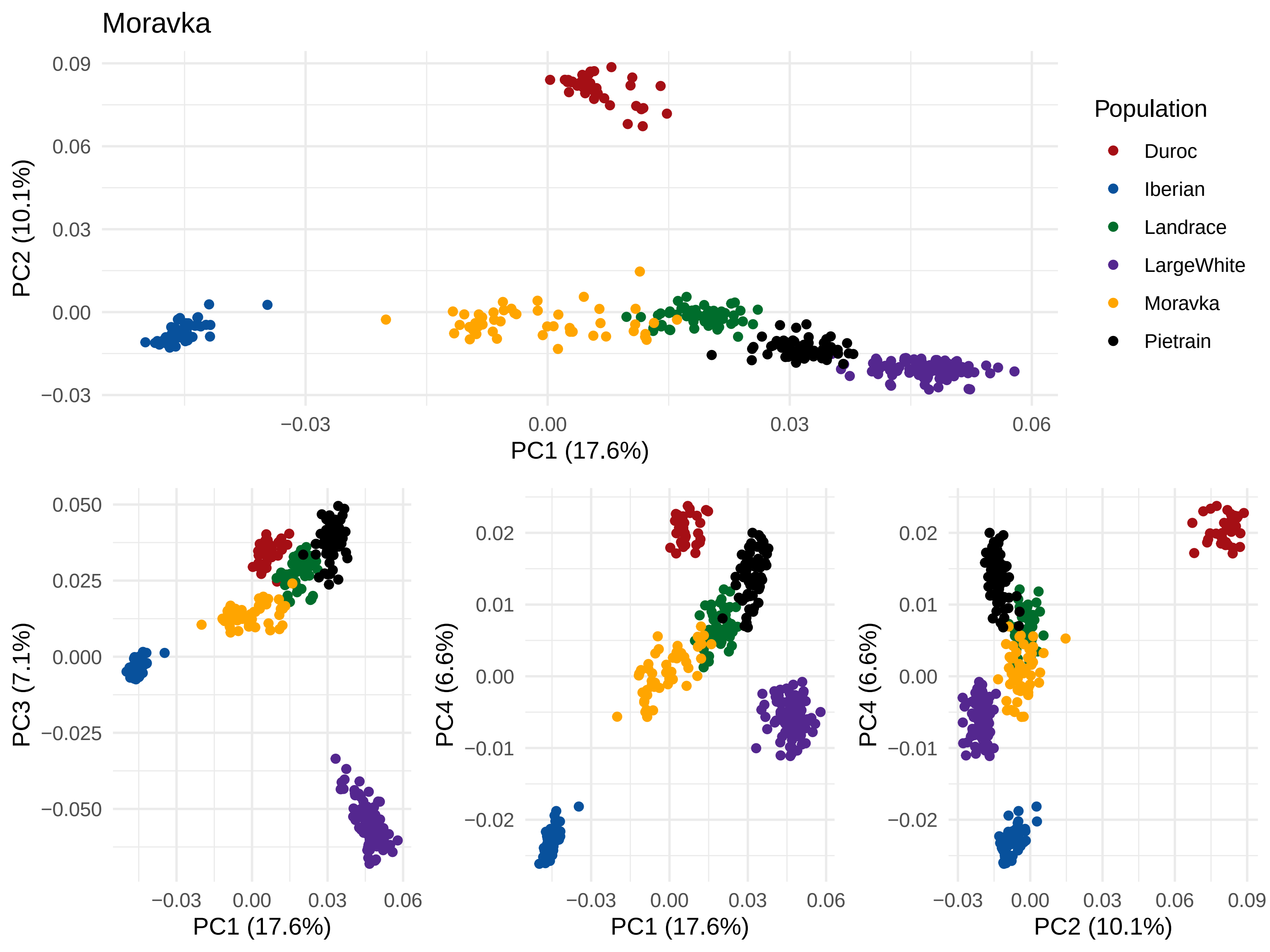

Moravka
PC2 (10.1%)
PC1 (17.6%)
0.09
0.06
0.03
0.00
−0.03
−0.03
0.00
0.03
0.06
Population
Duroc
Iberian
Landrace
LargeWhite
Moravka
Pietrain
PC3 (7.1%)
0.050
0.025
0.000
−0.025
−0.050
PC1 (17.6%)
PC4 (6.6%)
0.02
0.01
0.00
−0.01
−0.02
PC1 (17.6%)
PC4 (6.6%)
PC2 (10.1%)
0.09

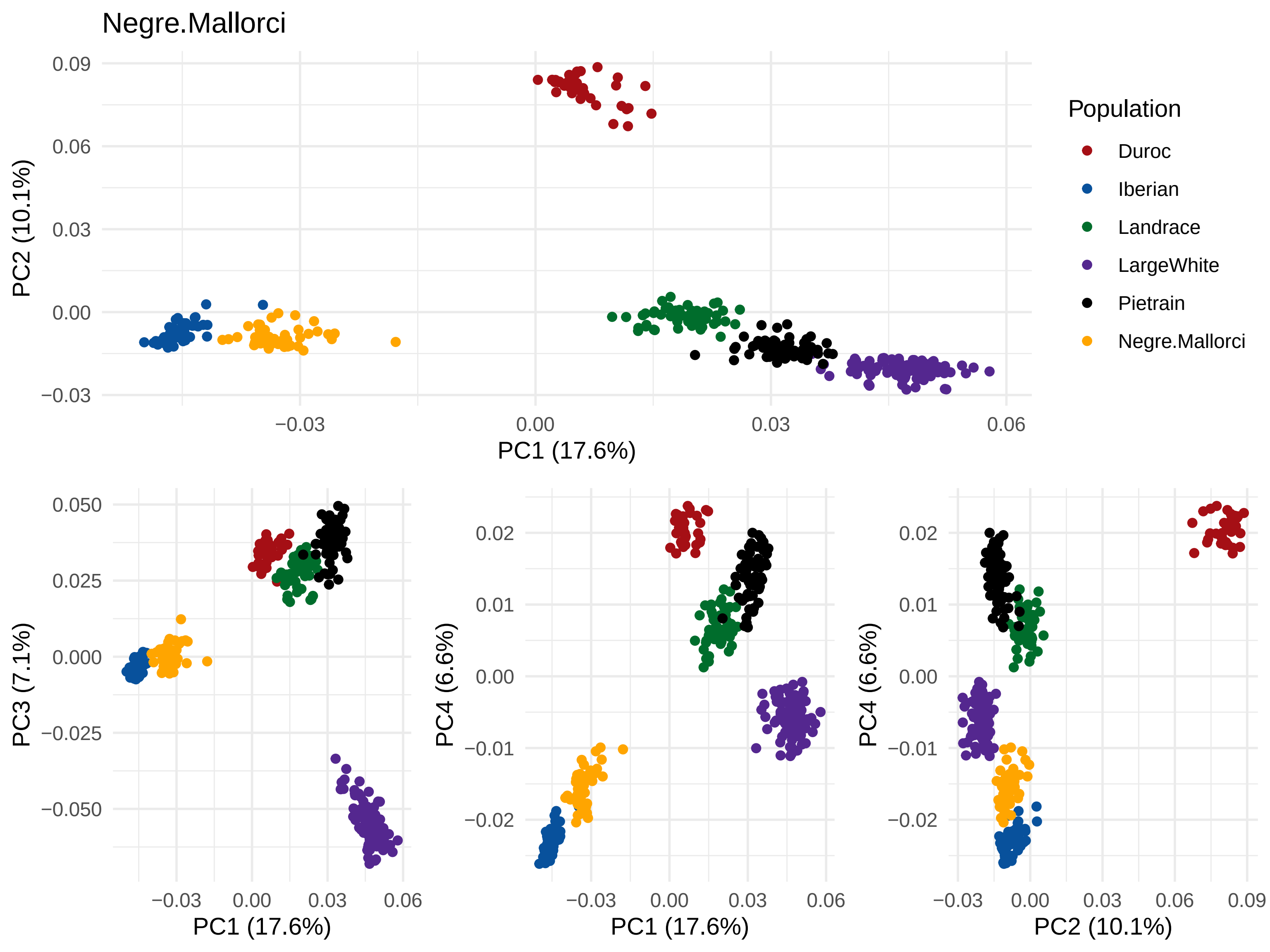

Negre.Mallorci
PC1 (17.6%)
PC2 (10.1%)
PC3 (7.1%)
PC4 (6.6%)
Population
Duroc
Iberian
Landrace
LargeWhite
Pietrain
Negre.Mallorci

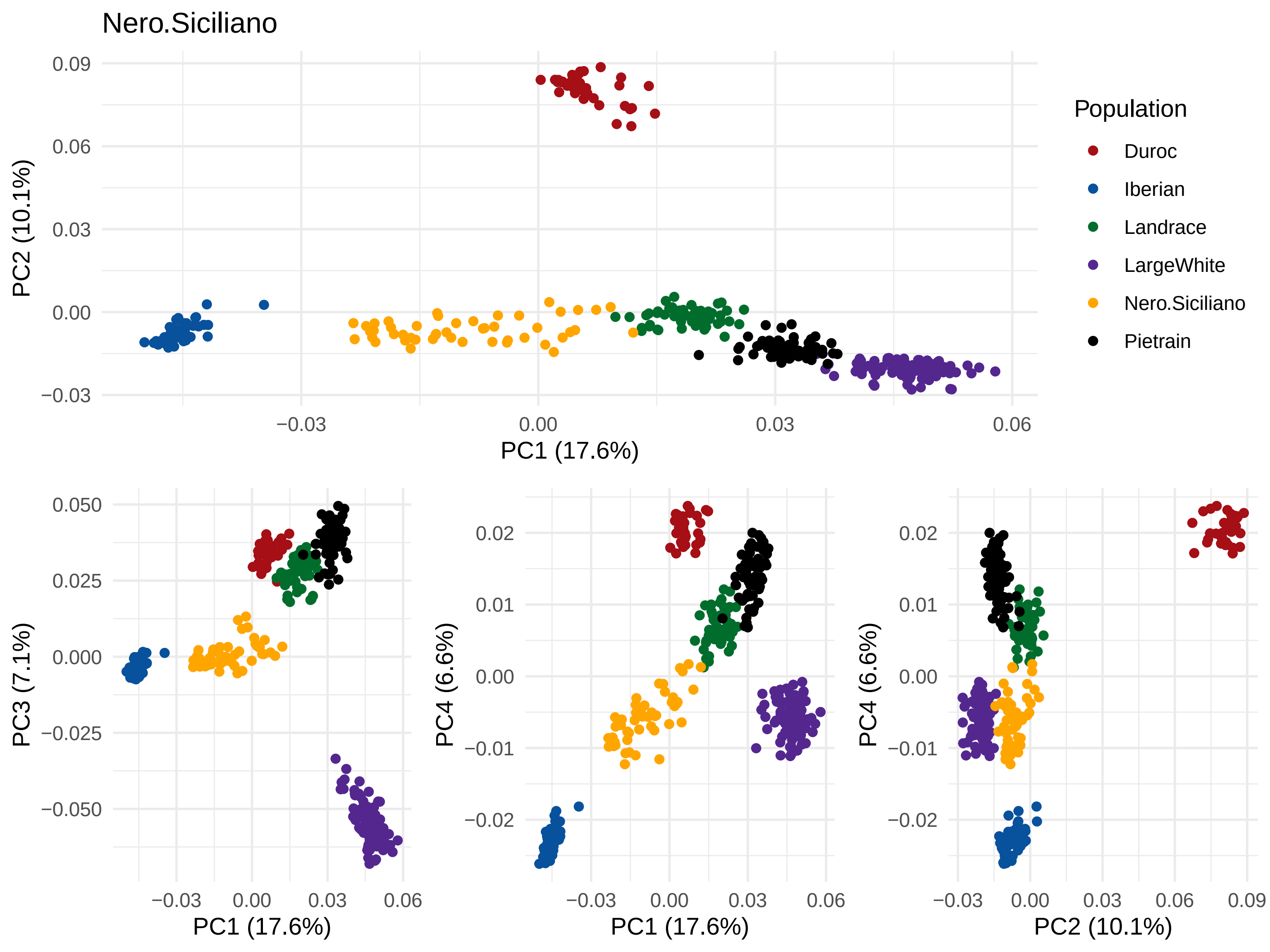
Nero.Siciliano
PC2 (10.1%)
PC1 (17.6%)
Population
Duroc
Iberian
Landrace
LargeWhite
Nero.Siciliano
Pietrain
0.09
0.06
0.03
0.00
−0.03
−0.03
0.00
0.03
0.06
PC3 (7.1%)
0.050
0.025
0.000
−0.025
−0.050
PC4 (6.6%)
0.02
0.01
0.00
−0.01
−0.02
PC2 (10.1%)
0.09

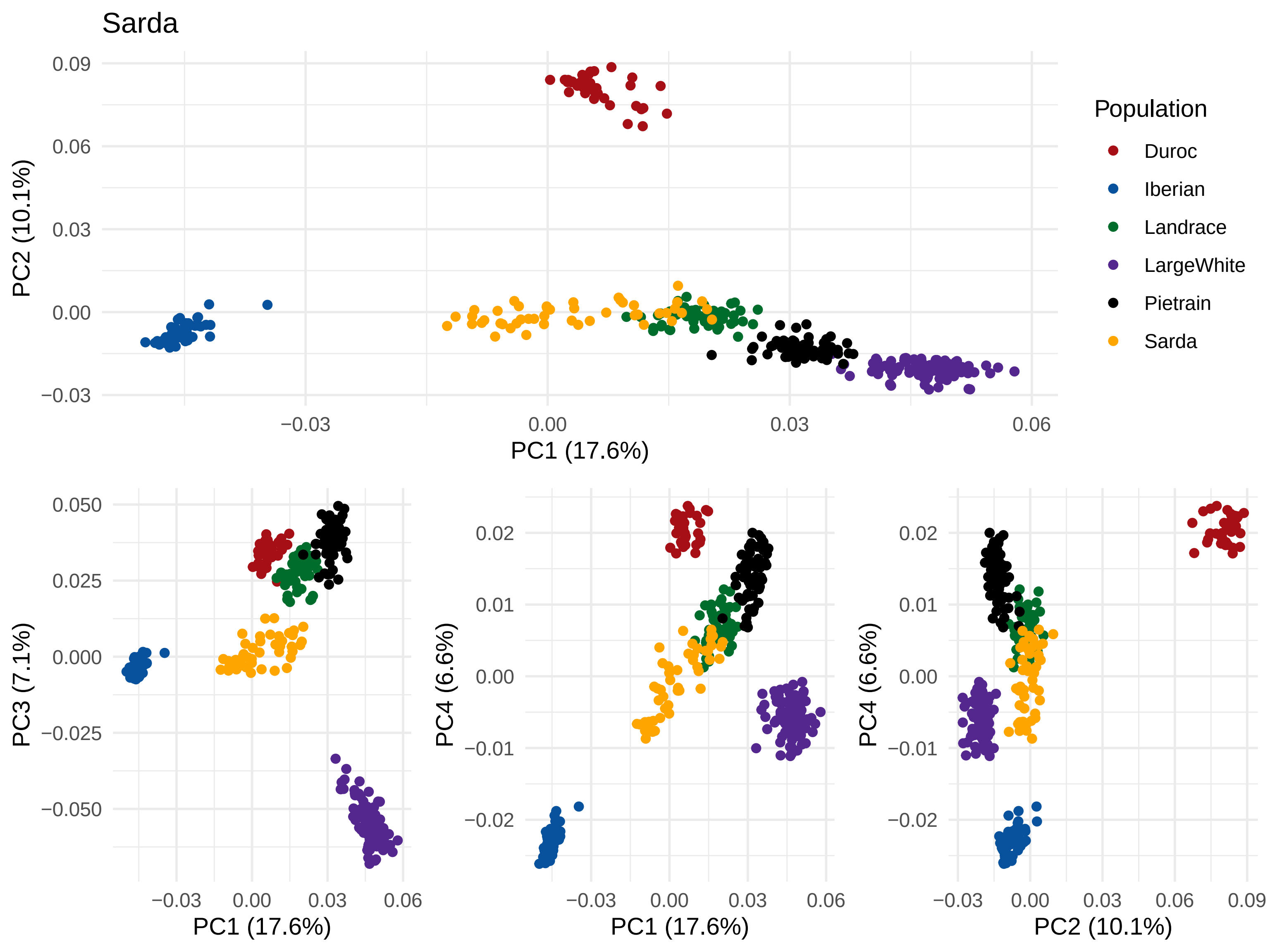

Sarda
PC1 (17.6%)
PC2 (10.1%)
PC3 (7.1%)
PC4 (6.6%)
Population
Duroc
Iberian
Landrace
LargeWhite
Pietrain
Sarda

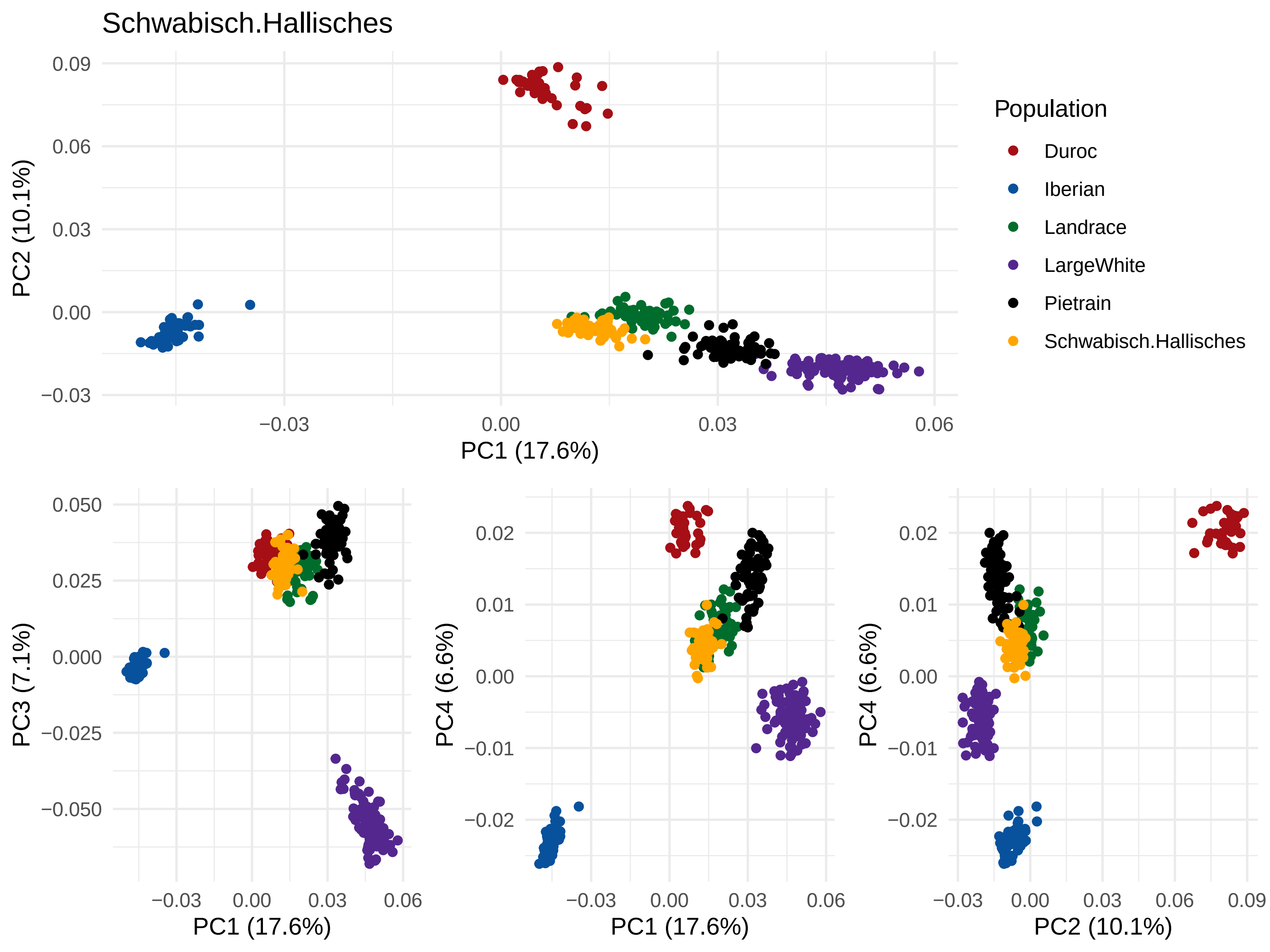
Schwabisch.Hallisches
PC1 (17.6%)
PC2 (10.1%)
PC3 (7.1%)
PC4 (6.6%)
Population
Duroc
Iberian
Landrace
LargeWhite
Pietrain
Schwabisch.Hallisches

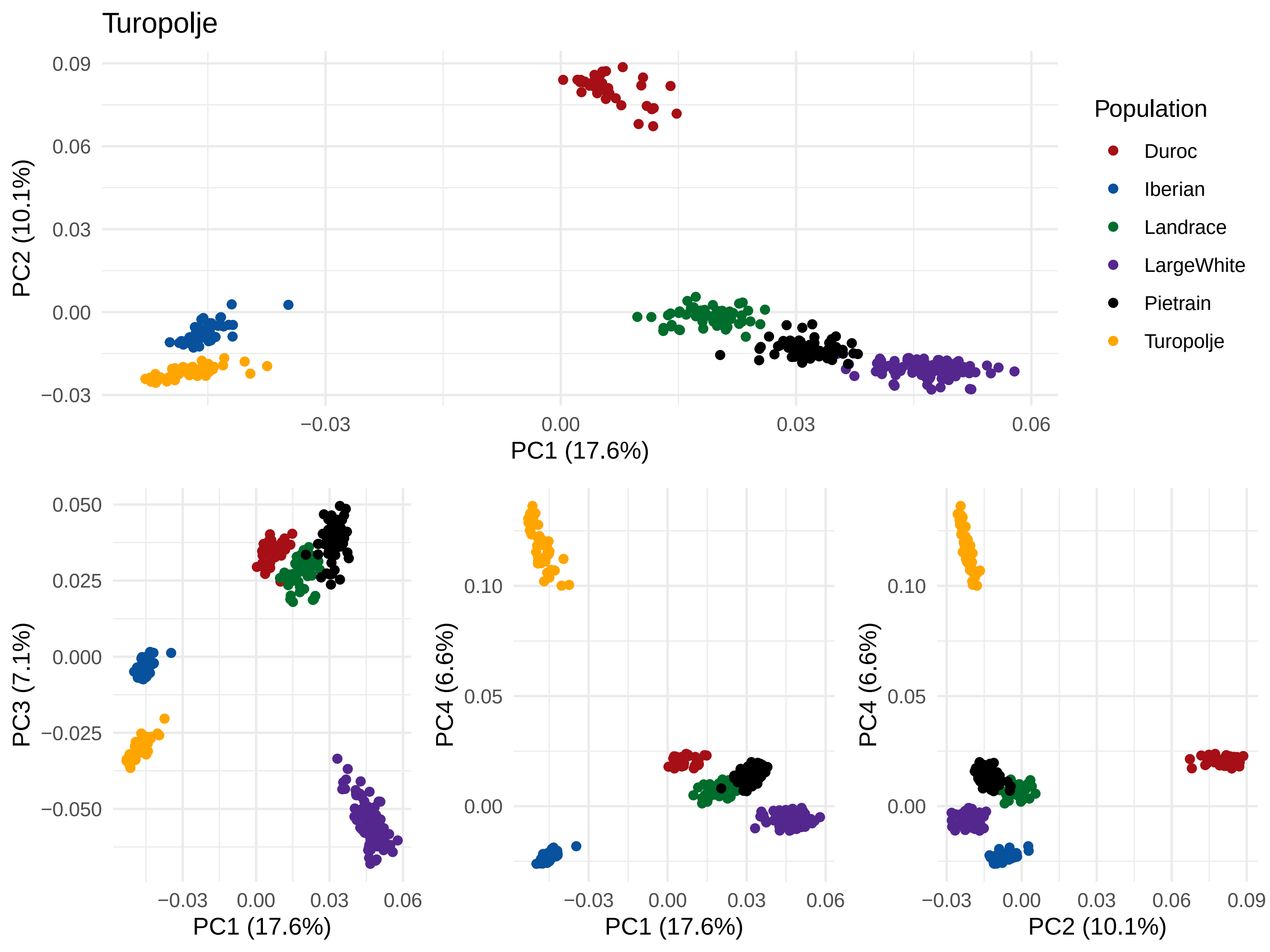

Turopolje
PC1 (17.6%)
PC2 (10.1%)
PC3 (7.1%)
PC4 (6.6%)
Population
Duroc
Iberian
Landrace
LargeWhite
Pietrain
Turopolje
